# Targeting p35/Cdk5 Signalling via CIP-Peptide Promotes Angiogenesis in Hypoxia

**DOI:** 10.1371/journal.pone.0075538

**Published:** 2013-09-30

**Authors:** Alessandra Bosutti, Jie Qi, Roberta Pennucci, David Bolton, Sabine Matou, Kamela Ali, Li-Huei Tsai, Jerzy Krupinski, Eugene B. Petcu, Joan Montaner, Raid Al Baradie, Francesca Caccuri, Arnaldo Caruso, Giulio Alessandri, Shant Kumar, Cristina Rodriguez, Jose Martinez-Gonzalez, Mark Slevin

**Affiliations:** 1 School of Healthcare Science, Manchester Metropolitan University, Manchester, United Kingdom; 2 Cell Adhesion Unit, Department of Neuroscience Dibit-Istituto Scientifico San Raffaele, Milano, Italy; 3 Chip-Man Technologies Ltd, Tampere, Finland; 4 Howard Hughes Medical Institute, Massachusetts Institute of Technology Picower Institute for Learning and Memory, Cambridge, Massachusetts, United States of America; 5 Stanley Centre for Psychiatric Research, Broad Institute of Massachusetts Institute of Technology and Harvard University, Cambridge, Massachusetts, United States of America; 6 Hospital Universitari Mútua de Terrassa, Department of Neurology, Barcelona, Spain; 7 Griffith University School of Medicine, Gold Coast Campus, Griffith University, Southport, Australia; 8 Neurovascular Research Laboratory, Vall De’Hebron University Hospital, Barcelona, Spain; 9 College of Applied Medical Science, Almajmaah University, Almajmaah, Kingdom of Saudi Arabia; 10 University of Brescia, Section of Microbiology, Department of Experimental and Applied Medicine, Medical School, Brescia, Italy; 11 Fondazione Istituto di Ricovero e Cura Carattere Scientifico Neurological Institute "Carlo Besta", Cellular Neurobiology Laboratory, Department of Cerebrovascular Diseases, Milan, Italy; 12 Department of Pathological Sciences, Manchester University and Christie Hospital, Manchester, United Kingdom; 13 Centro de Investigacion Cardiovascular, Hospital de la Santa Creu i Sant, Pau, Barcelona, Spain; INSERM, France

## Abstract

Cyclin-dependent kinase-5 (Cdk5) is over-expressed in both neurons and microvessels in hypoxic regions of stroke tissue and has a significant pathological role following hyper-phosphorylation leading to calpain-induced cell death. Here, we have identified a critical role of Cdk5 in cytoskeleton/focal dynamics, wherein its activator, p35, redistributes along actin microfilaments of spreading cells co-localising with p_(Tyr15_)Cdk5, talin/integrin beta-1 at the lamellipodia in polarising cells. Cdk5 inhibition (roscovitine) resulted in actin-cytoskeleton disorganisation, prevention of protein co-localization and inhibition of movement. Cells expressing Cdk5 (D144N) kinase mutant, were unable to spread, migrate and form tube-like structures or sprouts, while Cdk5 wild-type over-expression showed enhanced motility and angiogenesis in vitro, which was maintained during hypoxia. Gene microarray studies demonstrated myocyte enhancer factor (MEF2C) as a substrate for Cdk5-mediated angiogenesis in vitro. MEF2C showed nuclear co-immunoprecipitation with Cdk5 and almost complete inhibition of differentiation and sprout formation following siRNA knock-down. In hypoxia, insertion of Cdk5/p25-inhibitory peptide (CIP) vector preserved and enhanced in vitro angiogenesis. These results demonstrate the existence of critical and complementary signalling pathways through Cdk5 and p35, and through which coordination is a required factor for successful angiogenesis in sustained hypoxic condition.

## Introduction

The importance of angiogenesis in relation to neuronal replenishment and survival after stroke has been clearly demonstrated. In this respect, revascularization and associated reperfusion are vital determinants of tissue survival and patient recovery after stroke and therefore a major potential target for successful therapies [1]. Angiogenesis “*per se*” is a tightly regulated multi-step process, by which new blood vessels are formed from endothelial cell (EC) sprouts, emanating from pre-existing vessels and is mediated by the combination of a complex range of angiogenic and anti-angiogenic factors, driving the recruitment, migration, proliferation and differentiation of ECs [2]. Effective angiogenesis is strictly dependent on the proper spatial and temporal control of focal adhesions and activation of cytoskeletal organizers that regulate cell mechanical forces and the cell plasticity during migration, sprouting and cell differentiation within developing neo-capillary structures [3]. Beyond the typical canonical signalling molecules already characterized, a set of cyclin-dependent kinases (Cdks), has been shown to contribute significantly to the angiogenic response [4]. Among them, the Cdk5 is emerging as a potential important regulator of vasculogenesis [5].

Cdk5 is a proline-directed serine/threonine kinase highly expressed in the central nervous system. Initially due to the prevalence of its activity and activators (p35, p25 and p39) in neuronal cells, Cdk5 was considered as a neuron-specific kinase, involved in neurite outgrowth, neural migration, synaptic activity, neural cytoskeletal organization and neural survival [6]. Over the past decade, Cdk5 has been isolated in various non-neuronal tissues and cells [7], including ECs, and has been demonstrated to be essential for important cellular activities, including angiogenesis of Human Umbilical Vein ECs [5], neuronal cell adhesion and migration [7]; whereas its aberrant activation has been associated directly with neuronal cell death [8].

Increased expression of Cdk5 and of both p35 and p25 activators has been reported in human stroke [9,10] and after focal middle cerebral artery occlusion [8]. After brain ischemia, the rise in neuronal death is crucially associated by the Cdk5 hyper-activation mediated by p25 [8,9]. The excitotoxic signal transduction cascade induced by hypoxia leads to excessive activation of calpains, a group of calcium-activated cytosolic proteases, that cleave p35 to more stable p25 peptide, with the following Cdk5 hyperactivation and neuronal apoptosis; whereas modulation of p35 amount and its binding with Cdk5 confers cytoprotection [11,12]. Additionally, in our previous work, we have shown over-expression of Cdk5 together with p35/p25 in apoptotic brain ECs in hypoxic regions of stroked tissue [10]. We have shown the up-regulation and/or nuclear translocation of Cdk5 and p35 in neurons and ECs subjected to oxygen-glucose deficiency and their association with cellular damage as response to hypoxic conditions therefore, it is hypothesized that the Cdk5/p35 pathway may be a key pathophysiological determinant of ECs survival and ultimately outcome after infarction [13,10].

Currently, research focused on novel therapies directed to enhance angiogenesis, neurogenesis and improve neurologic function has highlighted the pharmacological inhibition of Cdk5/p25 signalling as a novel potential intervention point for cell protection and improved tissue remodelling after stroke [13,12]. However, since the use of some inhibitors would appear to affect also the physiological function of Cdk5 (i.e. Roscovitine [14]) and/or the regulation of synaptic plasticity (i.e. calpain inhibitors [15]), their introduction could lead to serious secondary side effects and thereby compromise any therapeutic efficacy [13]. Thus, identification and characterization of novel selective inhibitors of Cdk5/p25 signaling might produce a more viable potential therapeutic target [12] with particular emphasis to re-vascularization of the brain tissue [13].

Recently, the neuronal cyto-protective potential of a natural small peptide (CIP-peptide) was demonstrated after neurotoxic stress. CIP is a derived-p35 cleavage peptide, which selectively targets Cdk5/p25 activity without affecting Cdk5/p35 signalling [16], suggesting that CIP may represent a novel selective vascular protector/stimulator in hypoxic conditions such as those encountered after stroke.

In this study, we provide crucial evidence for a critical role of Cdk5 operating through distinct signalling mechanisms in the co-ordinated and sequential stimulation of cell spreading (actin fibres, talin and integrin-β-1) and tube differentiation and sprouting (MEF2C). Furthermore, we show that specific manipulation of Cdk5 signalling via administration to brain ECs of the CIP-peptide, allowed enhancement of in vitro cell migration with production of mature sprouts and tubes. Therefore, p35/Cdk5 manipulation may represent a novel strategy for EC protection in response to ischaemic conditions.

## Materials and Methods

### Cell culture and treatment with Roscovitine

Immortalized (hCMEC/D3) human brain microvascular EC cells (hBMEC; [17]), were kindly donated by Prof. Babette Weksler (Division of Haematology and Medical Oncology, Weill Medical College of Cornell University, New York). Cells were cultured on type I rat tail collagen (BD Bioscence Pharma, UK) coated plates, using microvascular EC medium-2 (EBM-2) from Clonetics (Lonza, Germany), supplemented with growth factors as recommended by the manufacturer. The concentration range (2.5-100µM) of (R)-roscovitine (Calbiochem, UK) were chosen based on other previously published studies demonstrating effectiveness [5]. 24h of incubation with roscovitine (50 µM) was found to be optimal for the study. The effects of (R)-roscovitine on cell proliferation (72h) were analysed using the CellTiter 96® AQueous One Solution Cell Proliferation Assay (MTS) (Promega Corporation, USA). Nuclear membrane damage and activation of cell apoptosis were evaluated by nuclear inclusion of propidium iodide counterstained with Hoechst 33258 (Sigma-Aldrich, Germany), and Quantikine® kit (R&D, USA) for active caspase-3 and DuoSet IC® kit (R&D, USA) for active p53, respectively. Cell treatment with staurosporine (Sigma-Aldrich), (0.5-1 µM) was used as positive control for apoptosis and cell damage analysis. Appropriate amounts of DMSO, the roscovitine vehicle, were used to ensure that the effects were roscovitine-specific.

### Cdk5-wt and Cdk5 kinase mutant stable transfections

Stable hBMEC transfectants for the wild type human Cdk5 (Cdk5-wt) or dominant-negative Cdk5 (N144) kinase inactive mutant (Cdk5-DN) were obtained from pcDNA3-cdk5GFP or pcDNA3-dnCDK5 expression vectors (Addgene, USA), using TransIT-2020 as transfection reagent (Mirus Bio LLC, Madison, USA). pcDNA3-GFP plasmid was used as a negative control (Addgene, USA). EGM-2 medium containing 500 µg/ml Geneticin (Invitrogene Life Science, UK) was used as selective media. Transfection efficiency was defined by the presence of GFP fluorescence and Cdk5 protein levels in different cell passages.

### CIP cloning and stable transfections

A modified EGFP-IRES vector containing CIP sequence [16], (pLV-CIP plasmids), was a kind gift from Prof. Sashi Kesavapany, National University of Singapore. CIP peptide is comprised of 126 residues of p35 protein (accession number: AAH26347.1) spanning from aa 154 to aa 279. CIP-PCR products from pLV-CIP plasmid, obtained by PCR (forward primer, TTA GGA TCC TTG CCT GGG TGA GTT TC and reverse primer, GTC GGA TCC TCA TGG GTC GGC ATT TAT C) and digested by BamHI (Invitrogen, UK) were then inserted by ligation into pcDNA3-GFP expression vector. After positive cloning in DH5α, purified pcDNA3-CIP-GFP vectors were stable introduced by transfection into hBMECs using TransIT-2020 transfection reagent (Mirus Bio LLC, Medison, USA). EGM-2 medium containing 500 µg/ml Geneticin (Invitrogene Life Science, UK), was used as selective media. Positive hBMECs clones were detected by GFP fluorescent signals and by the level of CIP mRNA by RT-PCR. *18S rRNA* (accession number: NM_022551.2) was used as housekeeping gene (forward primer, TAG AGG GAC AAG TGG CGT TC and reverse primer, TGT ACA AAG GGC AGG GACT T).

### In vitro capillary tubule-like structure formation on Matrige^TM^

The preparation of ECs and Matrigel^TM^ was performed as described previously [18]. 6x10^3^ cells were added to the Matrige^TM^ reduced in growth factors (Becton Dickinson, UK; prepared according to the supplier’s instructions), in complete EBM-2 media with or without scalar amount of tested compound. The cells were incubated for 24-72 h at 37°C. DMSO (vehicle) alone, at the same concentration as in the test samples, was also added in a group of controls in all experiments. The number of closed tubes was counted with a Nikon inverted microscope. All experiments were performed in triplicate.

### Spheroid based in vitro angiogenesis assay

The preparation of EC spheroids was performed as described previously [19,20]. Briefly, cells were harvested from sub-confluent monolayer cultures by trypsinization and 6x10^5^ cells were suspended in EGM-2 mixed with DMEM (Lonza, Germany) 10% FBS and 0.25% (w/v) carboxymethylcellulose (Sigma, UK). Cells were then seeded into non-adherent round-bottom 96-well plates to assemble into a single spheroid within 24 h at 37 °C, 5% CO_2_. Basic fibroblast growth factor (human recombinant FGF-2, (BD Bioscience, UK)) was used as positive control and added at a final concentration of 25 ng/ml. After 24h, gels were photographed and sprouting was assessed quantitatively.

### Colorimetric adhesion assay

The adhesion assay was performed in a 96-well pre-coated with collagen type-I and blocked with 2% BSA (w/v PBS) for 120 min at 37°C. Then the plates were heat denatured at 85°C for 10 min, to block non-specific bindings. As negative control, well were only coated with PBS. 1x10^4^ cells were seeded in each well and after 30 min from the seeding the non-adherent cells removed by washing with PBS. A value of 100% attachment was estimated by adding the cells to uncoated and fixed (methanol) plastic, without washing. After washing, 50ml of substrate solution (3.75mM p-nitrophenyl *N*-acetyl-β-d-glucosaminide, Sigma-Aldrich, 0.05M Sodium citrate pH 5.0, 0.25% Triton- X-100) was added to the wells and the plates incubated overnight at 37°C. After stopping the reaction (50mM glycine, pH10.4.5 mM EDTA) the optical density of each well was determined at 490nm using a microplate reader (Multiskan Ascent, Thermo Life Sciences, UK). DMSO (vehicle) alone, in the same concentration as was present in the test samples was also added in a secondary group of controls in all experiments. Experiments were done in triplicate.

### Spreading assay

Cells were placed in a 96-well plate and incubated for 1h. The plate was pre-treated as described for the colorimetric adhesion assay. The experiment was stopped by fixing the cells with cold methanol for 5 min at room temperature. Cells were then dried and stained with 1% methylene blue in water. Approximately 100 cells were examined in multiple microscopic fields. Multiple fields were imaged from each condition. Cells were classified as spread when flat and the cytoplasm visible around the entire circumference of the nucleus and un-spread, when round and without visible cytoplasm. The number of cells was then expressed as a % of total cells. Experiments were done in triplicate.

### In vitro angiogenesis assay with continuous live cell imaging

In different experiments, hBMECs, Cdk5-wt, Cdk5-DN, and/or CIP transfectants, (1-2x10^4^/ml), were added to a 24-well plate coated with collagen type I, in complete EBM-2 medium and maintained at 37 °C and 5% CO_2_ for 24 h to permit the formation of a confluent monolayer. Monolayer scratch wound with or without scalar amounts of the tested compound roscovitine (2-50 µM) was then induced by scoring the confluent cell monolayer with a sterile pipette tip to leave a scratch of approximately 0.4–0.5 mm in width. In vitro cell differentiation in tube like structures was performed seeding the cells (100,000 cells/ml) in growth media containing or excluding the roscovitine (50 µM) over a preformed gelled layer of Matrigel^TM^. In vitro adhesion, spreading and elongation assays were performed seeding separately non-confluent cells on to a collagen pre-coated 24-well tissue plate. Cells were maintained at 37°C and 5% CO_2_. The cell migration from the scratched monolayer and formation of tube like structures were monitored continuously in real time for 24h and 72h, respectively and cell adhesion and subsequent spreading for 2 h, using the automated cell culture Cell-IQ® Continuous Live Cell Imaging (Chip-Man Technologies Ltd, Finland). Cells were monitored via the ImagenTM program, and data analysed by AnalyserTM software. Each experiment was performed in triplicate. Hypoxia (1% O_2_, 5% CO_2_ and N_2_) experiments were performed using reduced EBM-2 (0.1% foetal bovine serum) media, with a pre-treatment of 24h. DMSO (vehicle) alone, in the same concentration as was present in the test samples was also added in a secondary group of controls in all experiments.

### Western Blotting

Medium was removed quickly and cells were washed twice with cold-ice PBS buffer. Then, protein extraction was performed using ice-cold RIPA buffer in the presence of a protease and phosphatase inhibitor cocktail. After 30 min of incubation in ice, total cell lysate was centrifuged at 12,000 g x 20 min at 4°C. Bredford protein assay (Bio-rad, UK) was used to quantify protein amount in cell lysate. Protein absorbance was determined at 580 nm using a microplate reader. 30 µg of protein was loaded and subsequently separated on 10% SDS-PAGE gel. Protein samples were transferred onto nitrocellulose membranes (Whatman International Ltd.), stained with amido-black solution and photographed to verify the equal loading and the quality of transfer. The membranes were subsequently incubated 1h with blocking solution (milk 1% in PBS-Tween buffer pH 7.4). Incubations with the primary antibodies against human Cdk5, MEF2C, Hsp70 (Abcam, UK), pTyr_(15_)Cdk5, p35, integrin beta-1 (Santa Cruz Biotechnology, UK), Cdk2 and pThr_(160_)Cdk2 (Calbiochem, UK), talin (Upstate, UK), and anti-GAPDH (Santa Cruz Biotechnology, UK) were performed with conditions as recommended by the manufacturers, overnight at 4°C. The membranes were then washed three time with PBS-Tween buffer before to be incubated with appropriate horseradish peroxidase conjugated secondary antibodies (Dako, UK) at room temperature. Protein bands were visualised using a chemiluminescence detection kit (Invitrogene Life Science) and protein contents were expressed as the ratio to GAPDH protein levels (Bio-Rad quantity one software). Analysis were performed in triplicate.

### Confocal and fluorescence microscopical analysis.

hCMEC/D3 cells were added to a glass cover slip coated with collagen type-I, in a 24-well plate in complete EBM-2 (2.5% fetal bovine serum) medium. After fixing with 4% paraformaldehyde (w/v in PBS) cell were washed with PBS and permeabilised with 0.1% Triton X-100 (v/v in PBS). After blocking with 1% BSA (w/v in PBS) incubations with primary antibodies against selected proteins were performed with conditions as recommended by the manufacturers, overnight at 4°C. Secondary antibodies Alexa Fluor 488 goat anti-rabbit IgG, Alexa Fluor 488 goat anti-mouse IgG, Alexa Fluor 568 anti-rabbit and Alexa Fluor 460 donkey anti goat (Molecular Probes/Invitrogen) were incubated with conditions as recommended by the manufacturer. Rhodamine-phalloidin (Molecular Probes/Invitrogen) was used to stain filamentous actin (F-actin). Nuclei were stained with Hoechst 33258 solution (Sigma, UK). Samples without primary antibodies were used as negative controls. Confocal microscopy was performed with a Leica TCS SPE1000 system and the accompanying Leica Application Suite Advanced Fluorescence (LAS AF) software (Leica Microsystems UK Ltd, Milton Keynes, Bucks, UK). Images were acquired at 1024x1024 pixel resolution. Cells were pre-treated with roscovitine (50 µM) for 24h or the appropriate vehicle (DMSO) and then seeded on the plates. Signal of autofluorescence was tested, including a negative control antibody (IgG) staining, as well. Experiments were performed in triplicate.

### Cdk5 kinase activity

Cell lysates for immunoprecipitates and kinase assay were obtained as described for western blotting, in contrast, a modified RIPA lacking ionic detergent was used. Cdk5 was immunoprecipitated using protein A-sepharose CL-4B and incubated in vitro with Histone H1 (Sigma, UK) and [γ-^32^P] ATP (Amersham Radiochemicals, UK). Before to start the immunoprecipitaiton, cell lysate was pre-cleared suing 100 µl of sepharose-A slurry per ml of cell lysate. The reaction mixture with Histone H1 was then resolved by SDS-PAGE followed by autoradiography. Active Cdk5 would result in the appearance on autoradiography film of a signal at the size of phosphorylated histone H1. A purified active complex Cdk5/p35 expressed in Sf21 cells (Millipore, Billerica, USA) served as a positive control. 2ng of the active complex was used in the assay. Immunoprecipitate using rabbit anti-goat antibody (Dako, UK) served as negative controls.

### MEF2C siRNAs

Knockdown of MEF2C expression was performed using Silencer® Select Pre-Designed & Validated siRNA (Applied Biosystems, Life technologies, USA). SiRNA delivery was performed using Lipofectamine 2000 (Invitrogene, UK). After three days of treatment, in selected media (EBM-2 with reduced FBS and without antibiotics), knockdown efficiency was tested via protein assay (Western Blotting) and cells used for in vitro angiogenesis analysis. *Silencer*® control scrambled siRNAs was used as a negative control. Assay conditions were performed as recommended by the manufacturers.

### Affymetrix Microarray

Total RNA was isolated using Trizol Reagent, and purified using the RNeasy Micro Kit (Qiagen, UK). The assay was performed according to the manufacturer’s instructions. Concentration and purity was determined using a NanoDrop ND-1000 spectrophotometer (NanoDrop Technologies) and RNA quality was assessed using Agilent RNA 6000 Nano Kit and 2100 Bioanalyzer platform (Agilent, USA). The expression pattern of untranfected cells and several clones from cells transfected with the empty vector or a dominant negative form of human Cdk5 were compared by microarray analysis. Microarrays and cDNA synthesis were performed following the GeneChip® WT cDNA Synthesis and amplification Kit (Affymetrix) and following the Affymetrix GeneChip® Whole Transcript (WT) Sense Target standard protocol [21,22]. The array data were summarized and normalized with the RMA algorithm using the Affymetrix Expression Console software. Statistical analysis was performed using Spotfire DecisionSite software (Integromics).

### Calpain activity

The fluorometric assay was performed according to the manufacturer’s instructions (Abcam, UK). Cytosolic proteins were specifically extracted without contaminations of cell membrane and lysosome proteases and preventing auto-activation of calpain during the extraction procedure according to the manufacturer’s instructions. Calpain activity was quantified using a fluorescence microplate reader BMG Labtech, USA. The fluorometric assay included a positive control of active calpain I, as well. Data were expressed as relative fluorescence units. Experiments were performed in triplicate.

### Statistical analysis

All data were expressed as mean ±SD (standard deviation). P values were calculated using the ANOVA one-way test using Student’s t-test for unpaired sample led by MS and followed by the Wilcoxon 2-sample test. Regression analysis was carried out according to standard methods provided by MS Excel and to SPSS version 12. Results were considered to be statistically significant at values of P <0.05.

## Results

### R-roscovitine is a selective inhibitor of Cdk5 activity in hBMECs

(R)-roscovitine is a well-recognized anti-neoplastic drug, carrying out both apoptotic [23] and anti-proliferative [24] actions with highly selective inhibitory potential on Cdk5 [5], and Cdk2 activities [25]. To ensure selective inhibition in our cellular model, we evaluated roscovitine effects on Cdk5 activity and on the protein levels of Cdk5, Cdk2 and activated pTyr_(15)_ pCdk5 and pThr_(160_)Cdk2. Cdk5 activity was estimated by measurement of histone H1 phosphorylation, one of its targets. The effect of scalar amounts of roscovitine on cell viability, cell apoptosis and proliferation were also determined. Roscovitine at the concentration of 50 µM inhibited completely Cdk5 activity (Figure S1) and reduced significantly Cdk5 and pCdk5 protein contents (Figure S1), without affecting Cdk2 or pThr_(160_)Cdk2 (Figure S1). Roscovitine did not affect cell viability, proliferation or apoptosis (Figure S2). Cytotoxic effects were seen at concentrations of 70 µM and greater.

### Cdk5 overexpression supports in vitro hBMEC angiogenesis during hypoxia

Recent observations suggest that apoptosis of neurons after brain ischemia is strongly associated with Cdk5 hyper-activation mediated by its activator p25 [9], however, normal signaling through the Cdk5/p35 complex promotes neuronal survival, whilst inhibition of normal Cdk5 activity may inhibit Hif-1α accumulation and concomitantly induce neuronal cell death [26]. Firstly, we optimized an *in vitro* model of low oxygen tension mimicking hypoxia during stroke, wherein hBMEC were exposed to 24h of low oxygen levels (1%). Hypoxia conditions were defined on the evidence that in human hypoxic brain tissue (i.e. after subarachnoid haemorrhage) the partial pressure of brain tissue oxygen (PtiO_2_) decreased dramatically from the normal values of 40 mmHg [27] to <10 mmHg [28]. Considering the conversion of % oxygen to units of mm Hg, that assumes 100% oxygen equal to 760 mm Hg, our system was set at 1% of O_2_ delivery, as previously described [10], to create a severe hypoxic environment [29].

**Figure 1:**
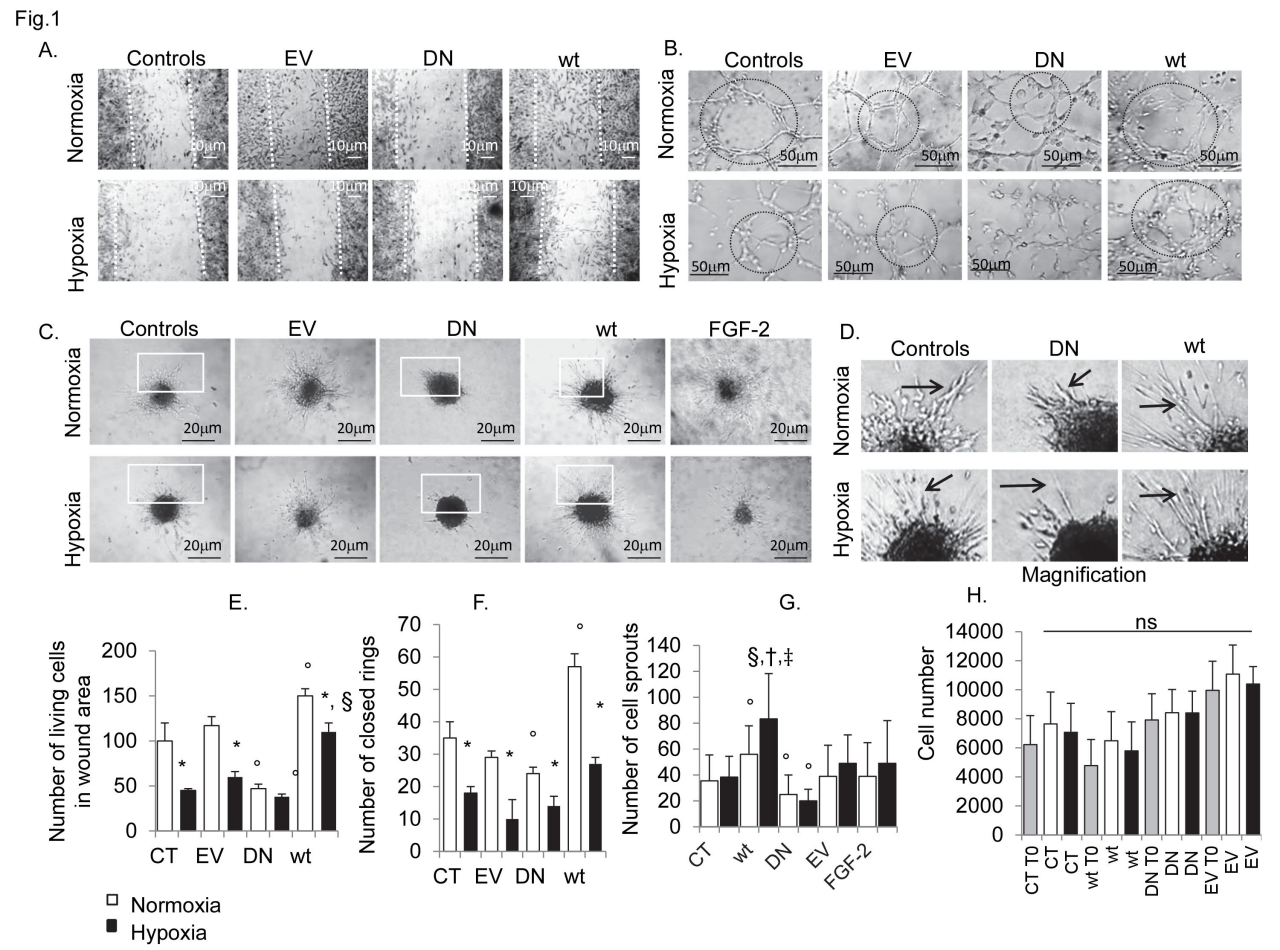
Consequences of hypoxia and Cdk5 deregulation on in vitro hBMECs angiogenesis. Phase contrast images showing the impact of hypoxia (24h 1% O_2_) and Cdk5 deregulation on cell migration (A), capillary tube formation (B) and spheroid cell sprouting (C). Assays were performed during 24h of hypoxia and/or normoxia-control condition, in stable hBMECs transfectants expressing Cdk5 wild-type (Cdk5-wt) and Cdk5 kinase inactive mutant Cdk5-(DN). Empty Vector (EV) transfectants served as negative controls of transfection. Hypoxia almost completely inhibited in vitro angiogenesis in hBMECs, as seen by the reduction in cell migration from scratched monolayer (A), tubule like structure formation (B) and/or cell sprouting (C). In normoxia, Cdk5-wt overexpression showed increased cell migration (A) and tubule formation (B), with an irregular formation of cell sprouts (C and D, arrows in magnification) which appeared more thin and disorganized, respect the controls. In contrast, Cdk5 kinase mutants (DN) were not able to migrate (A), to form new capillary structures (B) or sprouts (C, arrows in D). (G) The number of cell sprouts was markedly reduced. (A) Notably, in vitro angiogenesis was rescued in Cdk5-wt transfectants during hypoxia. (H) MTS assay was used to show the exclusion of other effects of hypoxia and transfection on cell proliferation. Calculated results are reported in graphs: E, number of living cells in wound area; F, number of closed capillary rings; G, number of cell sprouts; H, MTS assay showing cell proliferation in normoxic and hypoxic conditions, respectively. Data are expressed as mean ±SD of biological triplicates. * P <0.05 vs normoxia associated cell type, ° P <0.05 vs CT normoxia, † P <0.05 vs wt normoxia, § P <0.05 vs CT hypoxia, and ‡ P<0.01vs DN hypoxia; P value calculated using the Student t test. Bars in panel A, 10 µm. Each experiment was performed in triplicate.

**Figure 2:**
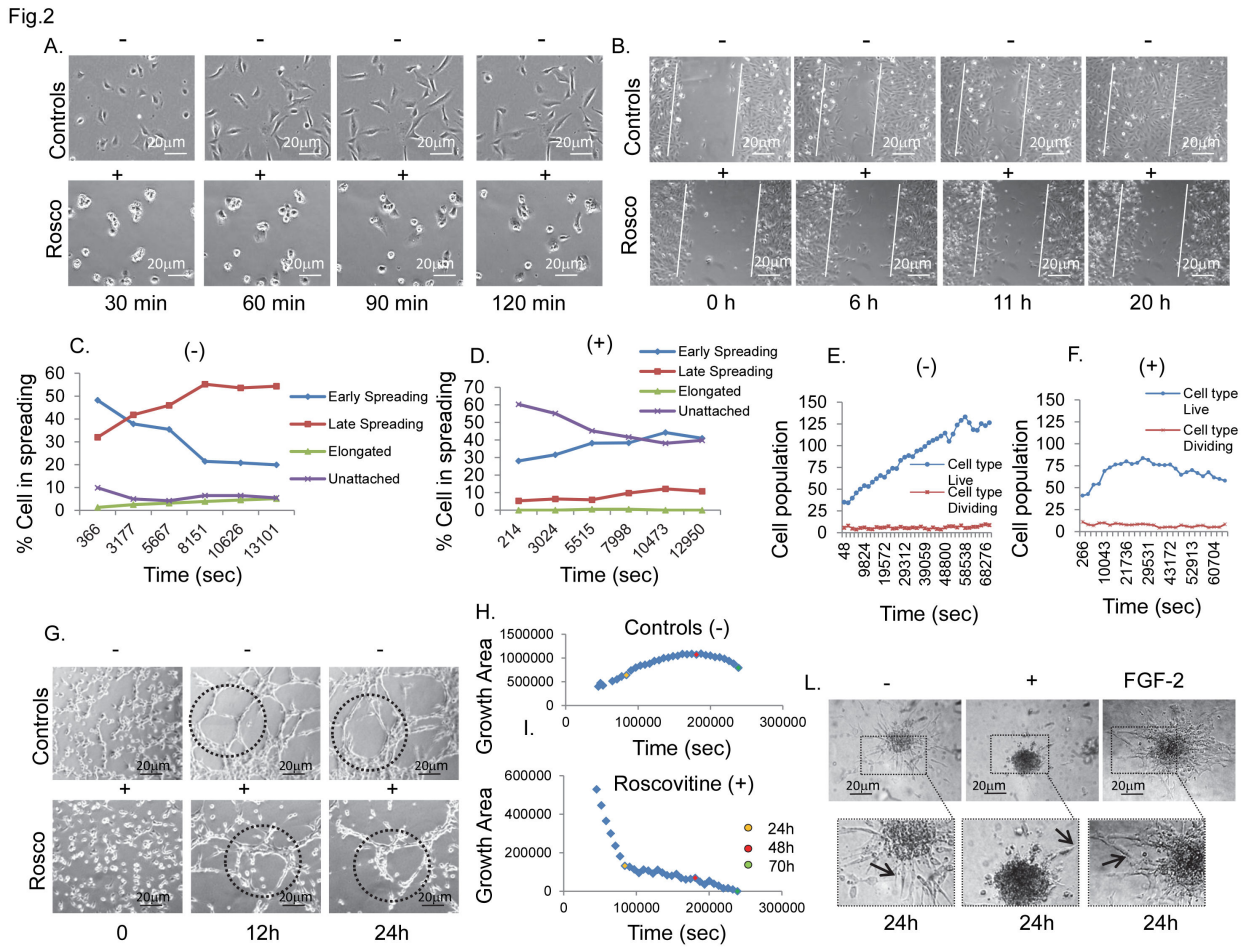
Cdk5 kinase activity is required for in vitro hBMECs angiogenesis. Figure shows phase contrast images of in vivo time course analysis of cell spreading (A), cell migration (B), and the formation of tubule like structure in roscovitine (50 µM) treated (+) and untreated (-) cells (G). Graphs C and D report the computational analysis of the characterization of cell typologies during the progression of different phases of cell spreading in controls (C) and roscovitine treated cells (D), determined using Analyser_TM_ software. Compared to the controls (C), roscovitine (D) inhibited cell elongation and increased the time dependent cell distribution between early and late spreading. Graphs E and F show the number of living and dividing cells presents in the wounded monolayer area in controls (E) and with roscovitine treatment (F) determined using Analyser_TM_ software. (G) Representative time-lapse images showing the progression of tubule formation throughout the time course, in controls and in treated cells. Graphs H and I, show the relative quantitative evaluation of growth area in controls (H) and treated cells (I), determined by Analyser_TM_ software. (I) Roscovitine reduced the growth area after 10h, mainly destabilizing neo-forming cell junctions and integrating cell networks. (L) Representative images showing the inhibitory effect of roscovitine on sprout formation, compared to controls (-) and FGF-2 stimulated cells (positive control). The phase contrast images have been acquired as z-stacks and with automated focusing. Bars, 20 µm. Original magnification in L, x 200. Each experiment was performed in triplicate.

In our model, the efficiency of hypoxia (Figure S3) was evidenced by the increased nuclear inclusion of propidium iodide (Figure S3), increased protein expression of heat shock protein Hsp70 (Figures S3B and S3F) and activation of calpain activity (Figure S3E). We found that hypoxia significantly reduced in vitro angiogenesis in hBMEC, reducing cell migration, tubule formation and/or cell sprouting. This was associated with decreased p35 protein content (Figure S3) and increased p25/p35 ratio (Figure S3), with no evident changes in Cdk5 expression (Figure S3). To understand the physiological importance of Cdk5/p35 signalling, Cdk5 activity was then deregulated using stable transfections of either Cdk5 kinase inactive mutant -D144N, (Cdk5-DN) or Cdk5 wild-type (Figure 1), and by pharmacological inhibition with roscovitine (Figure 2). The effects of Cdk5 inhibition on temporal and spatial cellular adaptations were then analysed by functional in vitro angiogenesis assays and monitored in real time using IQ Live Cell Imaging.

In normoxia, inhibition of Cdk5 kinase activity with roscovitine, resulted in a significant loss or inability to form and maintain potentially viable capillary structures (Figure 2), as shown by the inhibitory effect on in vitro cell adhesion (Figure S4), cell distribution between early and late spreading (Figure 2A), cell motility (Figure 2B), to culminate with the loss of EC tubule branching networks (Figure 2G) and cell sprouts (Figure 2L). Similarly, cells expressing the dominant Cdk5-kinase mutant D144N (Figure 1) were unable to spread, migrate and form new capillary structures or sprouts (Figure 1). Cells over-expressing Cdk5 wild-type (Figure 1) showed enhanced angiogenesis in vitro, characterized by increased cell motility, tubule and sprout formation. Noteworthy, in Cdk5-wt the increased angiogenic activity was accompanied by abnormal tubule and sprouts organization (smaller and thinner tubes and sprouts) (Figure 1C), suggesting that enhancing Cdk5 activity alone may not be a complete solution to allow protection and functional enhancement of in vitro angiogenesis. During hypoxia, inhibition of Cdk5 signalling was associated with a further inhibition of tube-formation beyond that produced by Cdk5-DN transfectants during normoxia (Figure 1), whereas cell migration, sprouting and tubule formation, were almost maintained at control-normoxic levels in the Cdk5-wt (Figure 1), thus Cdk5 overexpression preserved angiogenesis during in vitro hypoxia.

### pTyr_(15)_Cdk5 and p35 mobilization are essential for in vitro brain endothelial cell migration through association with integrin beta-1 and talin proteins

Cytoskeleton dynamic and focal complex organizations are crucial determinants for successful angiogenesis. We hypothesized that Cdk5 inhibition may affect the cell cyto-architecture and the observed inhibitory effects on angiogenesis in vitro may be the direct consequence of Cdk5 misplacement from actin and focal complexes. We also examined the involvement of the Cdk5 substrate, p35 in this cytoskeletal/focal organization.

**Figure 3:**
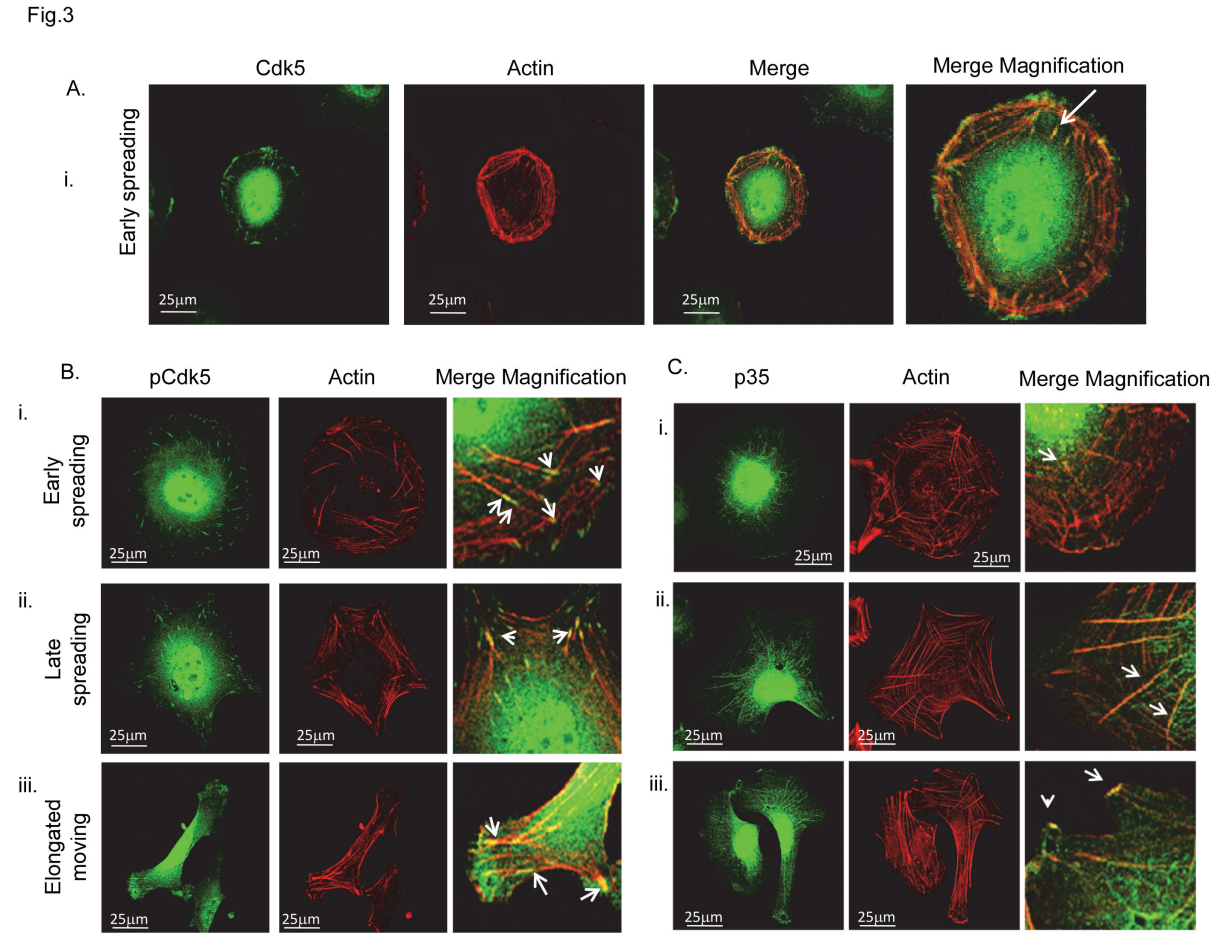
Cyto-architectural properties of Cdk5, activated Cdk5 (pCdk5) and p35. (A) Representative image showing Cdk5 localization at the tip of actin fibre (arrow) in early spreading (Ai). (B) Panel showing pCdk5 (pTyr_(Ser15)_) localization with actin tips (arrows) and fibre in early (Bi) and late (Bii) spreading and in moving (Biii) cells. (c) p35 co-localized with actin stress fibres and was distributed from the nucleus (Ci) as the cell spreads culminating in filament tips (arrows in merge) in moving (Ciii) and late (Cii) spreading cells. Immunofluorescence confocal microscope analysis. Objective 65x. Original magnification in merge, x 240. Bars, 25 µm. Original magnification in Ai merge, x 290. Original magnification in merge in panel A, x300. Each experiment was performed in triplicate.

**Figure 4:**
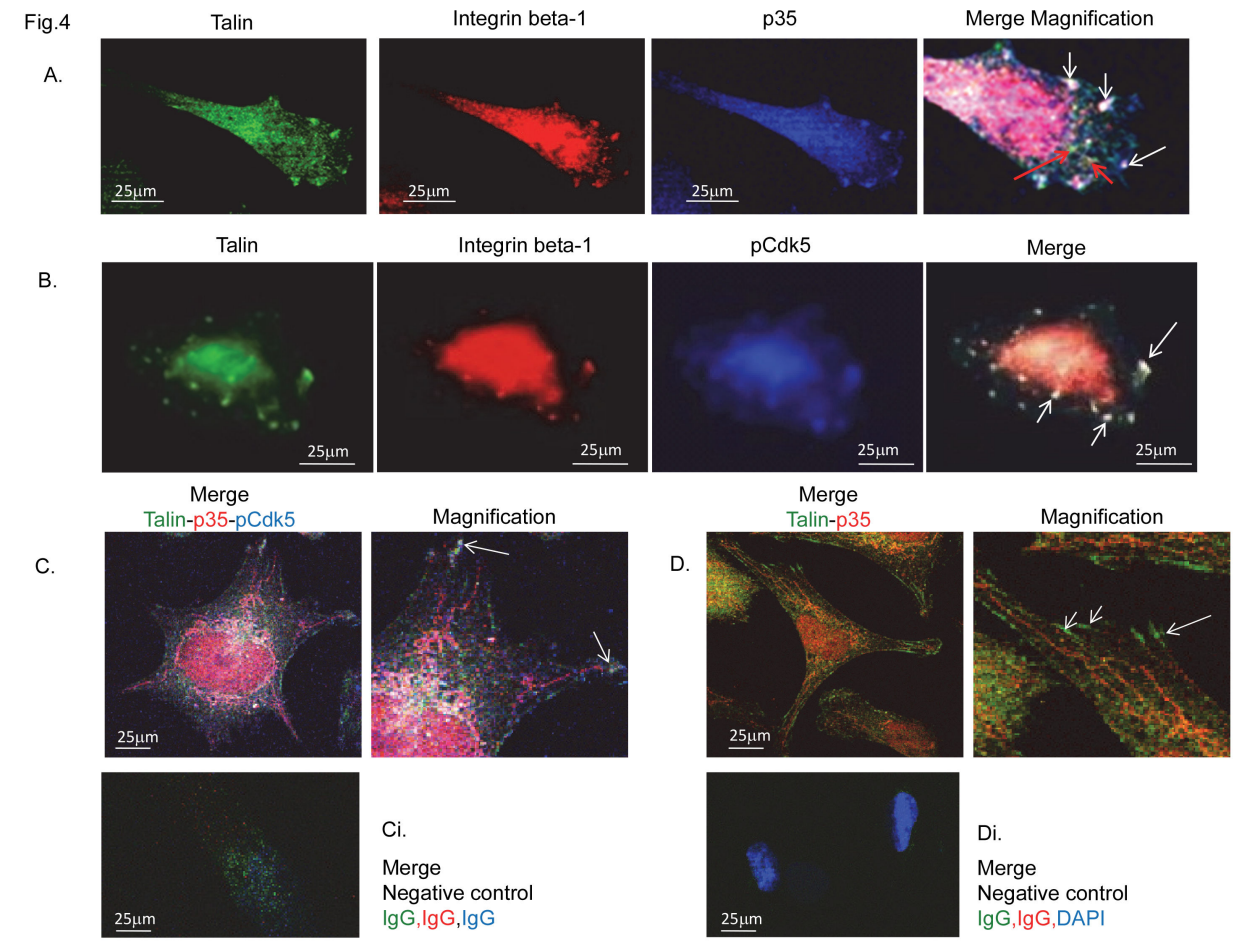
pCdk5 and p35 cluster with integrin beta-1 and talin into the lamellipodias. (A) Panel shows p35 clusters with talin and integin beta-1 into lamellipodia during cell movement. Red arrows in merge-magnification show dots of integrin and talin co-localisation. (B) Panel shows pCdk5 forming microvesicles with integrin beta-1 and talin at the lamellipodia in spreading cell. pCdk5 (C) and p35 (D) co-localized with talin in late spreading cells at focal tips (arrows in magnification). Immunofluorescent confocal microscopical analysis. Ci and Di show negative control for the immunoco-staining IgG-488 (green), IgG-568 (red), IgG-460 (blue) in Ci; and for the co-staining IgG-488 (green), IgG-568 (red), and DAPI (blue) in Di. Objective x40/x65. Original magnification in merge, x 240. Bars, 25 µm. Each experiment was performed in triplicate.

We found a marked and specific distribution of Cdk5, pCdk5 and p35 with F-actin in all phases of cell motility (Figure 3), having a precise time-dependent subcellular localization during spreading phases. Analysis of p35 intracellular localization determined at different time/stages of cell spreading, showed us that p35, emanating from the nucleus (Figure 3C), co-localized with actin stress fibres extending down the fibre from the nucleus as the cells spread, to culminate in filament tip expression in late spreading and moving cells (Figure 3C), suggesting a precise spatial-temporal mechanism linking p35 to stress fibre formation and/or contractility. Of particular note p35 (Figure 4A), and pCdk5 (Figure 4B), were clustered with talin and integin beta1 into lamellipodia during cell spreading (Figure 4C and 4D) and at the leading edge of elongated-moving cells, suggesting an involvement in the determination of cell polarity.

**Figure 5:**
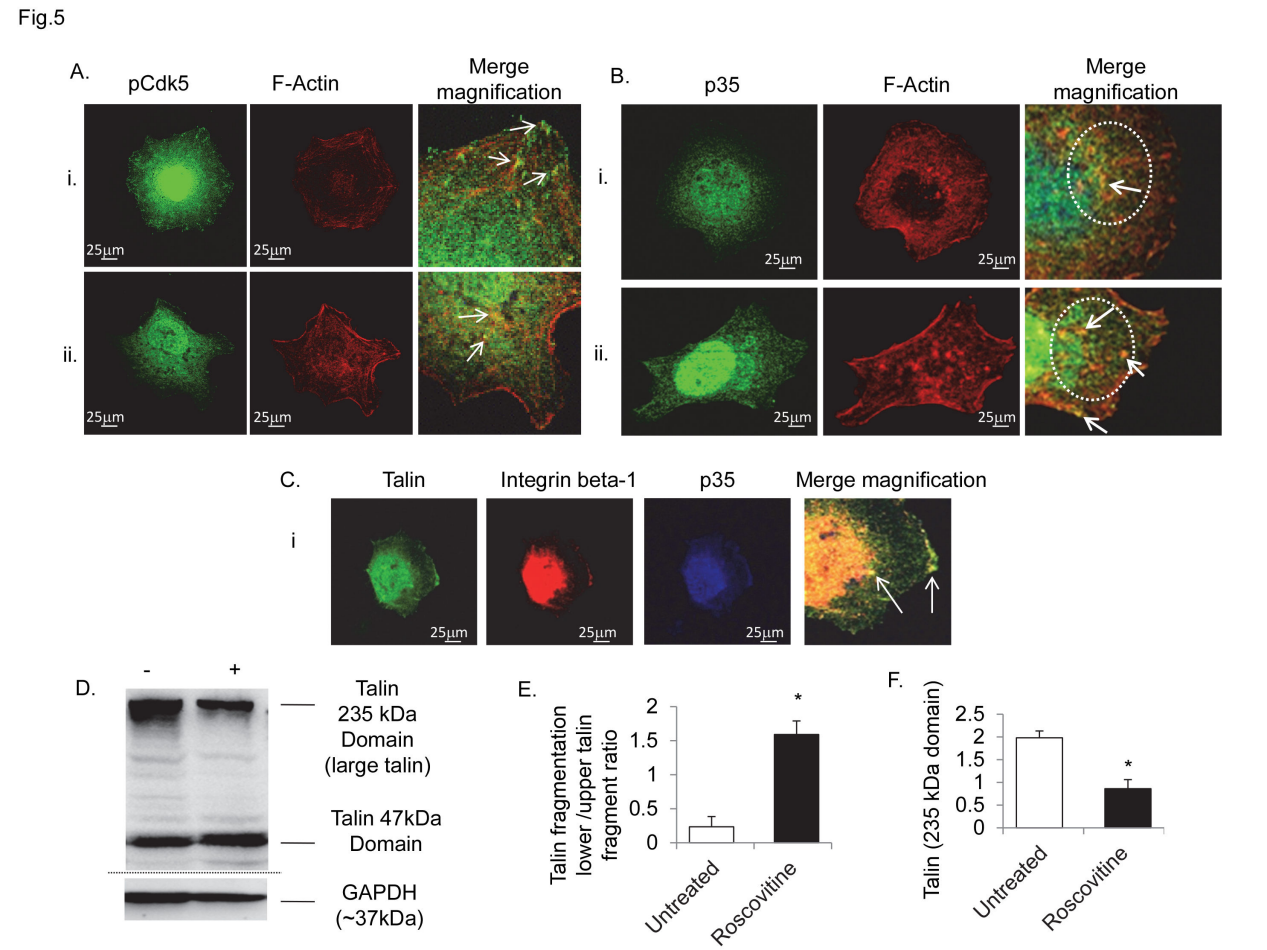
Blocking Cdk5 activity with roscovitine inhibited p35 and Cdk5 clustering with actin fibres and talin-integrin beta-1 complex. Inhibition of Cdk5 activity with roscovitine hampered co-localization of pCdk5 (panel A) and p35 (panel B) with actin filaments and stress fibres, resulting in production of abnormal cytoplasmic bundles with actin (arrows in magnification) in early spreading. (C) Roscovitine impaired the formation of talin tips and their co-localization with p35 and integrin beta-1. (D) Western blot showing increased talin fragmentation (47kDa domain), in roscovitine treated cells. Calculated results are reported in graphs: E, level of talin protein fragmentation in untreated cells and with roscovitine treatment; F, protein level of talin large domain (235kDa) in untreated and roscovitine treated cells. Data are expressed as mean ±SD of biological triplicates. * P <0.05. P value calculated using the Student t test. Immunofluorescence confocal analysis, objective 65x. Bars, 25 µm. Original magnification, x 200. Original magnification in merge Bii, x 230. Each experiment was performed in triplicate.

Inhibition of Cdk5 activity with roscovitine resulted in a total disorganisation of the actin- cytoskeleton and focal protein localization (Figure 5). With roscovitine, pCdk5 (Figure 5A) and p35 (Figure 5B) disassociated from actin filaments and stress fibres, to form, with actin, cytoplasmic clumping and bundles. Thus, p35 and Cdk5 activation seem essential for the mechanisms of actin polymerization. Inhibition of Cdk5 in Cdk5-DN mutant or roscovitine treated cells markedly increased talin fragmentation (Figure 5) and reduced the formation of focal talin tips (Figure 5C), preventing integrin beta-1/talin bundling (Figure 5D).

Therefore, we hypothesised Cdk5/p35 to have a multipart role where Cdk5 and p35 contribute to the mechanisms inducing matrix/focal cytoskeletal stability and on the other side, in those governing actin polymerization and depolymerization, as required for cell traction and progression of cell movement. In this scenario, p35 could be the/a stimulus for F-actin fibre polymerization and polarity leading to the formation of actin/focal plaque complexes. Inhibition of Cdk5 activity or reduced p35 content, as seen during hypoxia (Figure S3) may reduce cell tension, and consequently affects spreading architecture and in vitro migration.

### Cdk5 and MEF2C promote proper spatial organization of brain endothelial cells

The chaotic in vitro organization of EC tubule branches observed in Cdk5-wt transfectants, prompted us to speculate about the interplay of Cdk5 with other pathways involved in the promotion of EC differentiation. Using an affymetrix microarray (Figure S5) we analysed a cluster of genes involved in angiogenesis and the potential impact of Cdk5 deregulation on their expression. By Western blot analysis and co-immunoprecipitation assays, we identified the myocyte enhancer factor MEF2C (Figure S5), a transcription factor involved in cell differentiation and angiogenesis, as a putative molecular partner of Cdk5 in in vitro hBMEC/D3 angiogenesis (Figure S5).

**Figure 6:**
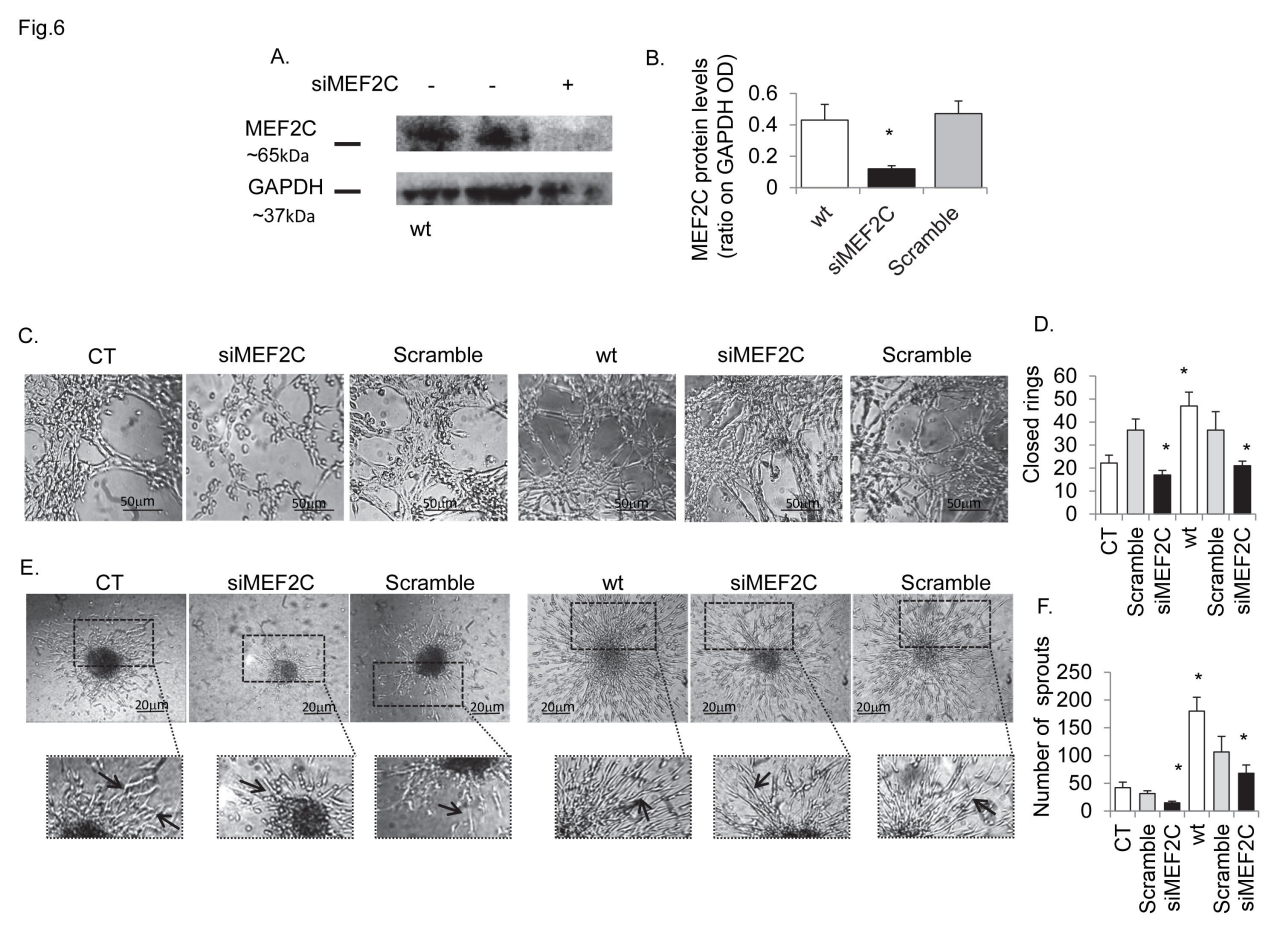
Cdk5 and MEF2C promote proper spatial organization of brain endothelial cells. Figure shows the effect of MEF2C knock down, using siRNAs on angiogenesis in control cells (CT) and Cdk5-wt transfectants (wt). Scrambled siRNA has been used as negative controls. (A) Western blot analysis showing the effect of siRNA on MEF2C protein levels in wt cells. (B) siRNA reduced MEF2C protein expression by approximately 80%. (C) Tubule network (24h) was inhibited in control cells, while appeared more structured in Cdk5-wt transfectants. Nonetheless, the number of complete closed rings (D) was significantly reduced in both controls and Cdk5-wt transfectants as the spheroid sprout length and thickness (E) or sprouts number (F). Calculated results are reported in graphs: B, MEF2C protein levels (as ration on GAPDH OD) in wt and siMEF2C, or scrambled wt transfected cells; D, number of closed capillary ring structure determined on Matrigel® assay; F, number of cell sprouts determined in spheroid assay. Data are expressed as mean ± SD of biological triplicates. For all figures * P <0.05 vs controls. P value calculated using the Student t test. Phase contrast images; bars, 20 µm; bars in panel C, 50 µm. Experiments were performed in triplicate.

Moreover, blocking Cdk5 activity in Cdk5-DN, notably reduced MEF2C protein levels and the opposite effect was seen in Cdk5-wt (Figure S5). In control cells, MEF2C knock down by siRNAs (Figure 6), reduced in vitro tubule formation (Figure 6C) and cell sprouting (Figure 6E), but had only a minor, non-significant effect on cell migration (Figure S6). In view of that, we did not find any effect of MEF2C knock down on actin fibre formation and/or on the co-localization of p35 with talin (Figure S6C) in moving cells. This could suggest that in hBMECs, MEF2C may be mainly required at the stage of differentiation. Accordingly, we observed that in Cdk5-wt (Figure 6C), MEF2C knockdown diminished the chaotic organization of capillary tubule like structure, demonstrating MEF2C as a Cdk5 molecular intermediate required to control the proper time and spatial capillary network in hBMECs. This interaction would likely take place at the nuclear level – since both proteins were observed to co-localise in the nuclei (Figure S5E).

### Modulation of Cdk5/p25 signalling rescued in vitro angiogenesis in acute hypoxia

The neuro-cytoprotective effect of CIP peptide has been amply demonstrated during neurotoxic stress, wherein the protection is conferred by the inhibition of Cdk5/p25 complex formation [16]. Thus, we asked whether the introduction of this peptide into brain ECs may prevent the detrimental effects induced by hypoxia, possibly reducing the cleavage of p35 into the more stable p25 peptide.

**Figure 7:**
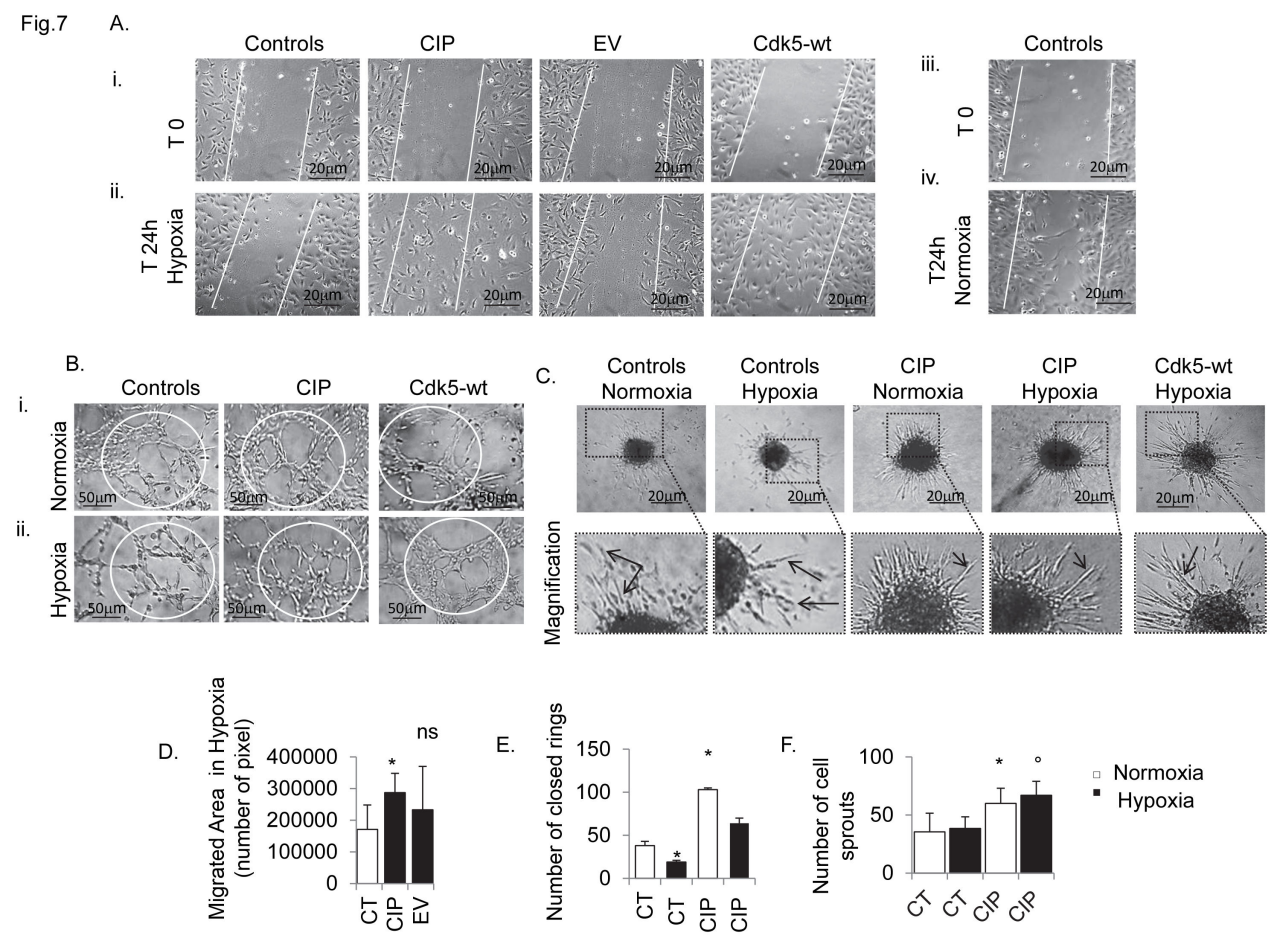
CIP-vector peptide rescues in vitro angiogenesis in hypoxia. (A) Panel reports a representative image of the effect of CIP–vector peptide on hBMECs cell migration during hypoxia (ii), versus controls in normoxia (iv), detected by phase contrast time-lapse analysis. (B,C) Representative contrast phase microscopy images of the effect of CIP–vector peptide on hBMECs tubule formation (B) and spheroid cell sprouting (C) in normoxic (i) and hypoxic conditions (ii). As Cdk5-wt CIP insertion significantly promoted cell migration during hypoxia, (D). (E) In CIP transfectants, the number of complete closed rings was significantly increased during normoxia (Bi), and maintained during hypoxia (Bii). Compared to the controls and Cdk5-wt, CIP insertion promoted the formation of spheroid–sprouts, which appeared with thicker lumens (C) and increased in number (F) in both hypoxic or normoxic conditions. Calculated results vs. controls and EV are reported in graphs: D, number of migrated cells in wound area during hypoxia, determined by Analyser_TM_ software; E, number of closed capillary ring structures on Matrigel® assay; F, number of cell sprouts in spheroid assay. Data are expressed as mean ±SD of biological triplicates. For all figures * P <0.05, and ° P <0.01. P value calculated using the Student t test. The phase contrast images of migration assay have been acquired as z-stacks and with automated focusing. Bars, 20µm; bars panel C, 50 µm. Bars, panel A 20 µm; panel B, 50 µm; 20 µm in panel C. Each experiment was performed in triplicate.

**Figure 8:**
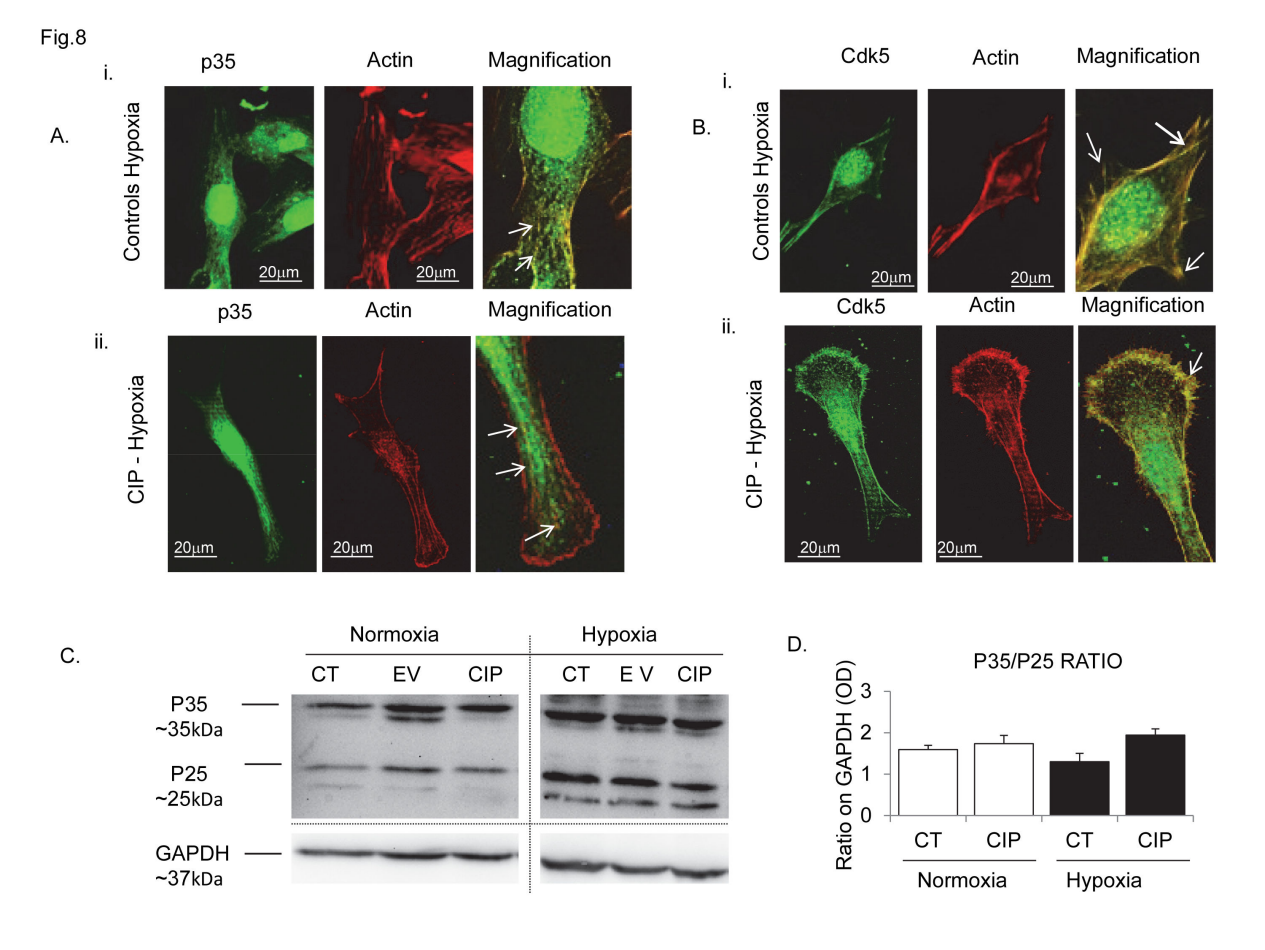
CIP-vector peptide preserves p35/actin intracellular co-localization during hypoxia. (A) Images show the effect of hypoxia on p35 (left panel, Ai) and Cdk5 (right panel, Bi) intracellular distribution with actin filaments and fibres in hBMECs. Under hypoxia, p35 (Ai) lost its canonical filament-like structure organization (in merge, upper left panel) and localization at actin fibre tips, showing a more diffuse cytoplasmic distribution, while Cdk5 (Bi) maintained its co-localization with actin fibre tips (arrows in merge, upper right panel) (ii). Compared to the controls CIP insertion protected p35 (Aii) and Cdk5 (Bii) intracellular localization with actin filaments and fibres from the effects of hypoxia, this was associated with increased p35 protein contents (C) and increased p35/p25 protein ratio (D). Calculated results are reported in graph D; optical density (OD) of p35/p25 protein ratio in control untransfected cells and in CIP transfectants determined in normoxic and hypoxic conditions. Typical images from n=2 independent experiments. Immunofluorescence confocal analysis; bars, 20 µm. Original magnification, x 100. Each experiment was performed in triplicate.

We found that CIP expression (Figure S7) was able to rescue cell migration (Figure 7A) and the in vitro angiogenic potential during hypoxia, increasing the capillarization (Figure 7B) and the formation of new sprouts (Figure 7C). At the cytoskeletal level the organization of stress fibres with p35 clustering, which was de-regulated during hypoxia in control cells (Figure 8Ai), was markedly stabilized (Figure 8Aii). This was accompanied by an increased p35/p25 protein ratio (Figure 8, C and D), indicating that CIP potentially can interfere with/inhibit p35 cleavage induced by hypoxia (Figure S3C). Notably, Cdk5 localization with actin fibres was only marginally affected by hypoxia (Figure 8Bi) or in CIP transfectants (Figure 8 Bii). CIP vector insertion also demonstrated a positive in vitro pro-angiogenic effect during normoxic conditions, promoting cell migration (Figure S8) and increasing talin content, specifically with production of a greater number of talin tips containing p35 protein (Figure S9).

## Discussion

Cdk5 hyper-activation mediated by the cleaved p35 fragment, p25, and initiated via calpains is crucially associated with neuronal cytoskeletal derangement, neuronal apoptosis and cell death during ischemic brain insult [9], while activation of Cdk5 through the parent p35 is associated with cytoprotection [11]. Cdk5 and p35/p25 are concomitantly overexpressed in ECs in hypoxic regions of stroke tissue [10], suggesting that the balance of signaling between these pathways may help to define cellular fate in relation to angiogenesis or cell protection after stroke [13]. Our study demonstrates that Cdk5 activation through its major substrate p35 provides a key trigger for initiation and maintenance of in vitro brain EC angiogenesis and that this interaction and signalling cascade is crucial for ensuring correct cytoskeletal organisation/dynamics, involving integrin beta-1 and talin, and allowing cell spreading and subsequent cell migration. Operating via the novel binding partner, nuclear transcription factor MEF2C, angiogenesis in vitro proceeds with cell differentiation and tube-like-structure formation. Our results also show that introducing a natural inhibitor of p25/Cdk5 complex formation, the CIP peptide [16], we were able to alter the balance of p25/p35-Cdk5 signalling in favour of p35 and concomitantly and significantly increase in vitro brain EC angiogenesis during normoxia and most importantly, promoting cell survival and capillarisation in hypoxic conditions.

Several studies in animal and in vitro models have described important functions of the myogenic transcriptional effector MEF2C, a crucial pro-survival protein, during vascular development, vasculogenesis and angiogenesis [30,31]. Targeted deletion of the MEF2C gene in mice, results in embryonic lethality with significant cardiovascular defects and severe vascular abnormalities, wherein endothelial cells retain the capability to differentiate but are unable to organize into a functional vascular network [32]. Here, we found a significant association between MEF2C protein levels and degree of in vitro angiogenesis using both Cdk5-wt and DN-kinase mutant transfectants. An increase in MEF2C expression in Cdk5-wt resulted in abnormal and chaotic vascular network production, higher levels of cellular migration and longer but thinner spheroid–sprout lumens. In contrast, the reduced amount of MEF2C seen in DN-kinase mutants was coupled with significantly reduced and disorganised EC spatial organisation.

Knockdown of MEF2C using small-interfering RNAs in Cdk5-wt, re-established the proper cellular network, whilst reducing abnormal cell migration and sprouting. In non-transfected cells MEF2C knockdown blocked almost completely the formation of neo-capillaries, suggesting that MEF2C could be an important Cdk5-signalling intermediate in brain ECs, and a key requirement for functional in vitro angiogenesis. A relationship between Cdk5 and MEF2C in angiogenesis has not been reported previously. However, Cdk5/p25 complexes have been shown to phosphorylate other members of MEF2 family (MEF2A and MEF2D), inhibiting their pro-survival activity, through a caspase-3 dependent degradation, in response to neurotoxic stress [33,34], without show any targeting on MEF2C [33]. Notably, restoration of the active form of MEF2 has been proved to be protective following focal ischemia [35] and increasing MEF2 phosphorylation levels (ser59) has been recently described in rat brain ischemic stroke models after inhibition of calpain/Cdk5/p25 signalling, by sildenafil, used to antagonise chemical hypoxia induced by malonate [12].

Here, using a protein pull down assay, we were able to co-immunoprecipitate the two proteins, but whether this association may be due to a regulatory mechanism on MEF2C stability mediated by Cdk5 phosphorylation is still to be defined. Further work should investigate whether Cdk5 is acting as a direct regulator of MEF2C gene expression, perhaps via the E2F1-pRb axis [36,37].

Published work has demonstrated non-nuclear functions linked to Cdk5 in the regulation of cytoskeleton dynamic and motility [5], showing Cdk5 as a key modulator of talin stability and therefore essential to cell migration [38]. Thus, we investigated the interplay of Cdk5 and p35 with classical molecular players of the control of mechanical forces and motility. We found a specific time/spatial interaction of active (phosphorylated) p-Cdk5 and p35 with integrin beta-1 and talin, directed to the formation of microvesicles in late-spreading and elongated/moving cells. Accordingly with previous observations [5,39,40], p35, Cdk5 and pCdk5 localized at the focal tips of actin stress fibres. Inhibition of this interaction, by roscovitine or in DN-mutants blocked in our cellular model, cell protrusion and stable adhesions. Notably, we found that p35 localization alongside actin fibres during spreading is time-dependent and culminates with the formation of focal clusters with Cdk5, integrin beta-1 and talin. On this basis, we hypothesised that p35 may be a putative adaptor implicated in the generation of cellular tension. The association of p35 with talin and integrin beta-1 may result in the formation of a molecular bridge among cytoplasmic domains of integrins and actin fibres [38]. p35-mediated activation of Cdk5 at that sites, in conjunction with talin, may be required for activation/phosphorylation of integrins and to the resulting actin polarised organization [40]. Hence, we suggest that the formation of p35 complexes with pCdk5 at focal adhesion points may relate to Cdk5 activation at focal-regions and be involved in integrins kinase/activation.

In our study, hypoxia markedly impaired in vitro angiogenesis, leading to a noticeable alteration in stress fibre cyto-structure with delocalization of p35 from these fibres. This was reflected in a marked inhibition of cell spreading and migration. These changes were associated with decreased p35 expression and a parallel increase in calpain expression and in the cleavage product p25. Notably, modulating Cdk5/p35 activation with the novel natural inhibitor of p25/Cdk5 complex, the CIP- peptide, we were able to reduce p35 cleavage to p25 and ameliorate angiogenesis during in vitro hypoxia. CIP increased recovery of cell spreading and formation of stable neo-capillaries, potentially increasing p35 localization with actin fibres and therefore the stabilization of the actin cytoskeleton. On this base, we propose that p35/cdk5 signalling elicits a specific protective role during in vitro angiogenesis and together with the observed protective action of the CIP peptide, may potentially aid in tissue remodelling after ischaemic stroke.

In conclusion, our results show that the p35/Cdk5 is a pathway that is critically involved in controlling cell dynamics of brain EC. Activation of p35/Cdk5 signalling at the expense of p25-Cdk5 plays a protective role in hypoxic conditions, and positively contributes to preserve cell motility and the proper spatial and temporal control of cytoskeletal dynamics, which is essential for successful angiogenesis.

## Supporting Information

Figure S1 **R-roscovitine selectively inhibited Cdk5 activity and tyrosine phosphorylation: (A-B) Inhibition of histone phosphorylation; (C-E) Cdk5 phosphorylation but not that of Cdk2.** Each experiment was performed in triplicate.
(TIF)

Figure S2 **R-roscovitine was not cytotoxic to hBMECs at concentrations used: (A-B) no increase in propidium Iodide inclusion was found, whilst (C-D) neither p-53 nor caspase-3 activity increased.** (E) No reduction in cell number was seen in proliferation assays up until 70 µM concentration of R-roscovitine. All experiments were performed three times.
(TIF)

Figure S3 **In vitro hypoxia induced cell damaging effects in hBMECs: (A and D) increased Propidium Iodide nuclear inclusion; (B) increased expression of Hsp70; (B-C) increased p-35 protein cleavage and more p25 expression; (E) an increase in calpain expression, and (F-G) an increase in the ratio of p25/p35.** All experiments were carried out three times.
(TIF)

Figure S4 **R-roscovitine inhibited, spreading, migration and tube formation of hBMECs: (Ai and C) R-roscovitine showed a reduced number of cells with the capacity to elongate as well as closure in a scratch wound assay (Aii and D) and form closed tube-like structures (Aiii and E).** Numbers of cells attaching in cell culture plates was also significantly reduced (B). All experiments were repeated three times.
(TIF)

Figure S5 **Gene microarray studies identified MEF2C down-regulation in Cdk5-DN mutants: (A) gene array results with Western blot confirmation of MEF2C protein down-regulation in DN mutants (B-C); (D) immunoprecipitation showing direct intracellular binding of Cdk5 with MEF2C protein and (E), double immunoflourescent labelling demonstrating MEF2C co-localization with phospho-Cdk5.** All experiments were repeated three times.
(TIF)

Figure S6 **siRNA down-regulation of MEF2C inhibited hBMEC angiogenesis and talin-p35 co-localization in spreading cells: (A-B) siRNA to MEF2C significantly inhibited hBMEC migration in the scratch wound assay; (C) double immunoflourescent labelling showed notably reduced talin-p35 protein interaction at the tips of spreading cells concomitant with reduced ability to polarise and spread.** All experiments were performed three times.
(TIF)

Figure S7 **CIP transfectants expressed higher levels of CIP peptide: (A) immunofluorescent identification of CIP-GFP cellular uptake; (B-C) CIP transfectants e.g. CIP14 and CIP 17 expressed notably more peptide.**
(TIF)

Figure S8 **Effect of CIP transfection on hBMEC wound healing: (A-B) The presence of CIP-vector inside hBMEC significantly increased wound closure/migration compared with empty vector/control cells.** Experiments were repeated three times.
(TIF)

Figure S9 (TIF)
