# Supplementary figures and images for: Targeting p35/Cdk5 Signalling via CIP-Peptide Promotes Angiogenesis in Hypoxia
Source: PLoS One. 2013 Sep 30;8(9):e75538. doi: 10.1371/journal.pone.0075538 (PMC3787057; doi:10.1371/journal.pone.0075538)

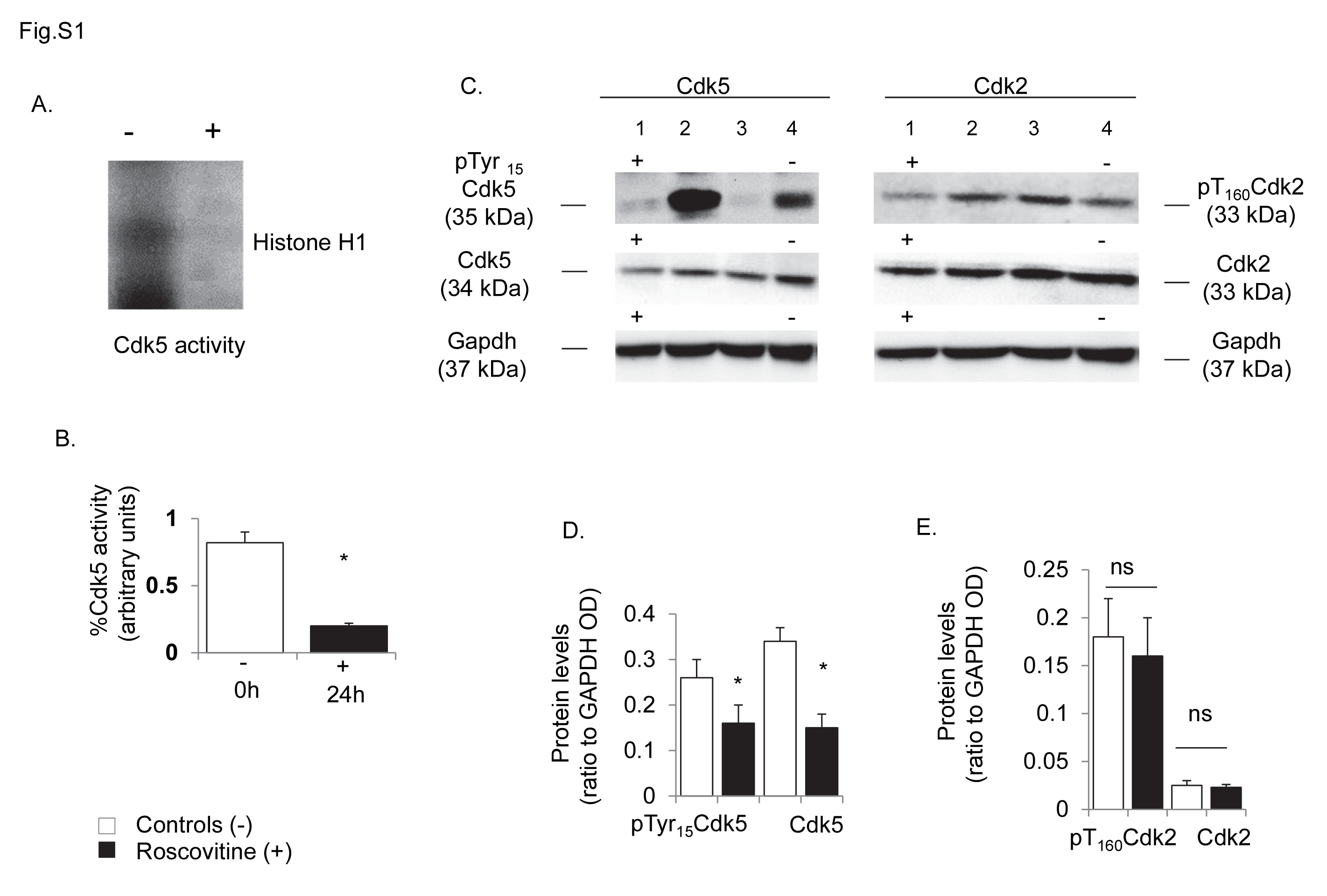

Supplement: Figure S1 — R-roscovitine selectively inhibited Cdk5 activity and tyrosine phosphorylation: (A-B) Inhibition of histone phosphorylation; (C-E) Cdk5 phosphorylation but not that of Cdk2. Each experiment was performed in triplicate. (TIF) [file pone.0075538.s001.tif]

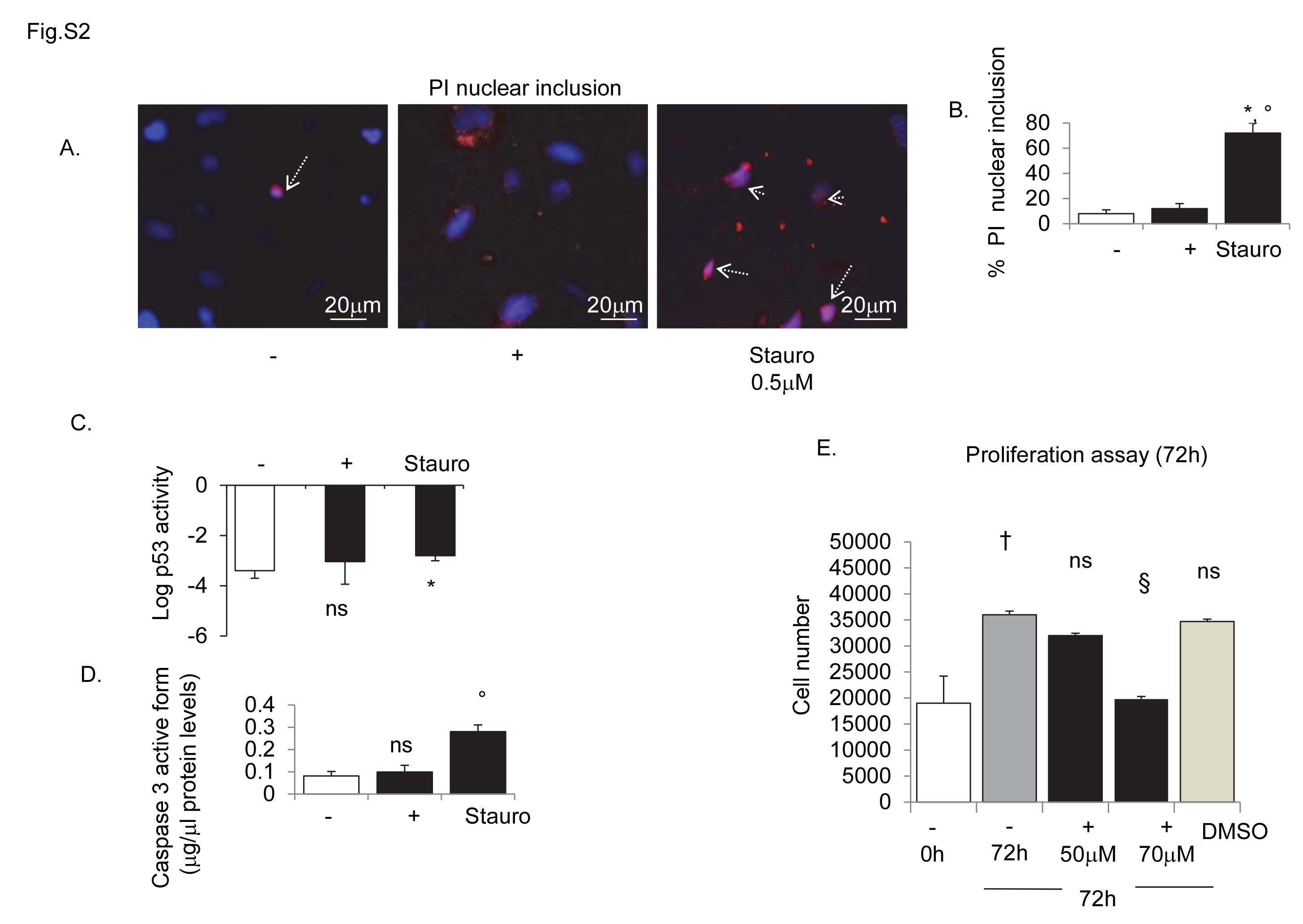

Supplement: Figure S2 — R-roscovitine was not cytotoxic to hBMECs at concentrations used: (A-B) no increase in propidium Iodide inclusion was found, whilst (C-D) neither p-53 nor caspase-3 activity increased. (E) No reduction in cell number was seen in proliferation assays up until 70 µM concentration of R-roscovitine. All experiments were performed three times. (TIF) [file pone.0075538.s002.tif]

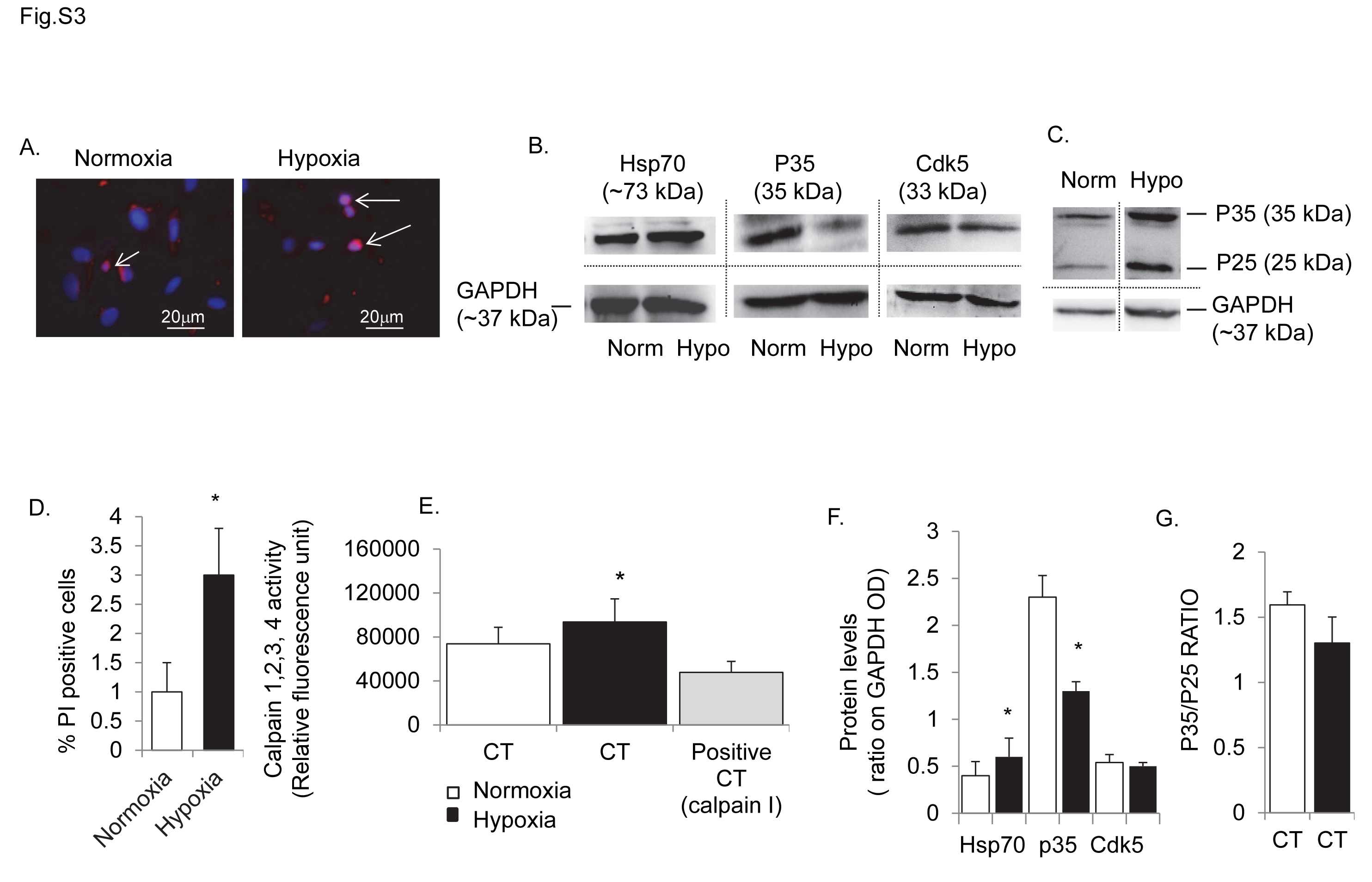

Supplement: Figure S3 — In vitro hypoxia induced cell damaging effects in hBMECs: (A and D) increased Propidium Iodide nuclear inclusion; (B) increased expression of Hsp70; (B-C) increased p-35 protein cleavage and more p25 expression; (E) an increase in calpain expression, and (F-G) an increase in the ratio of p25/p35. All experiments were carried out three times. (TIF) [file pone.0075538.s003.tif]

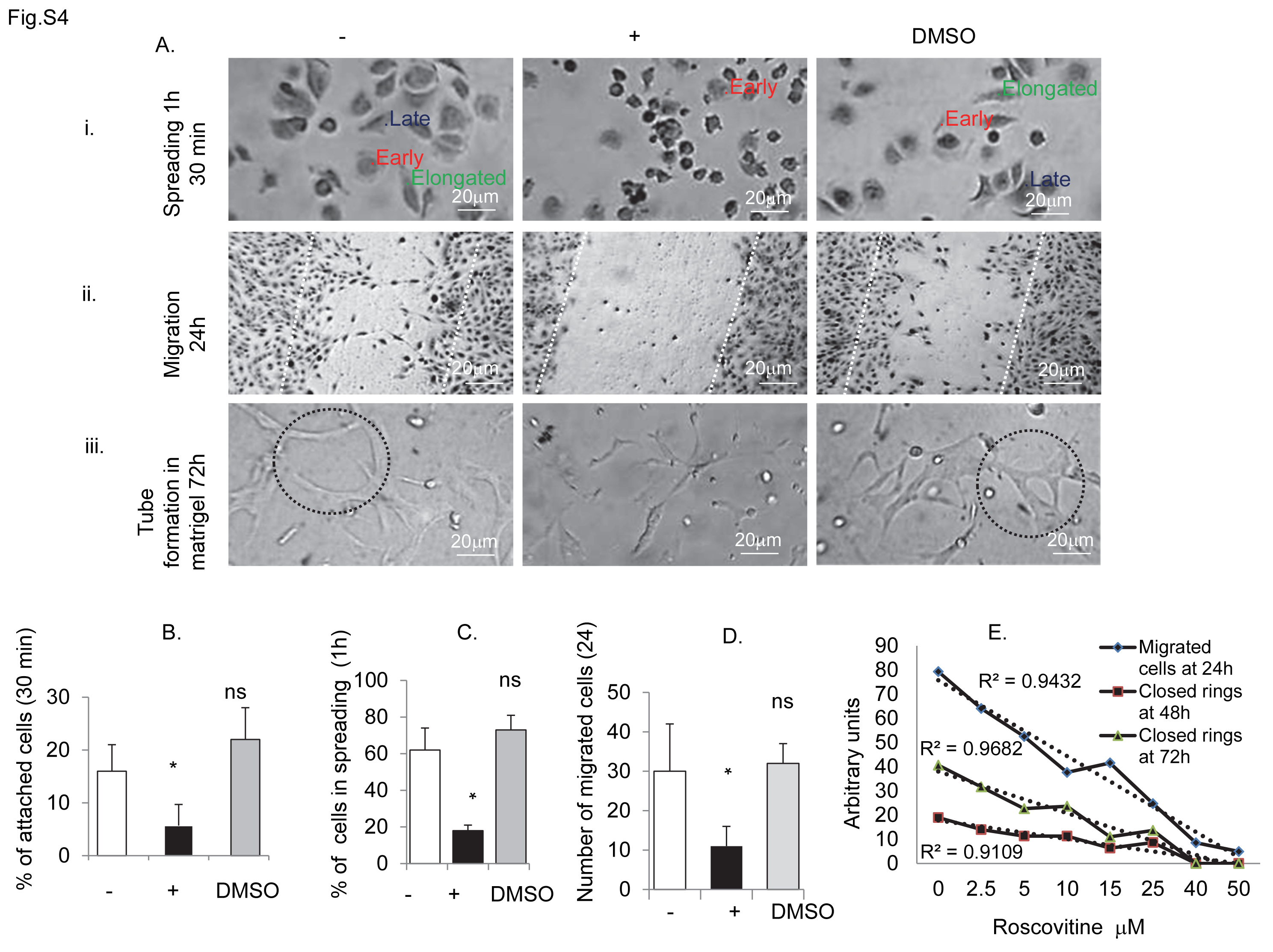

Supplement: Figure S4 — R-roscovitine inhibited, spreading, migration and tube formation of hBMECs: (Ai and C) R-roscovitine showed a reduced number of cells with the capacity to elongate as well as closure in a scratch wound assay (Aii and D) and form closed tube-like structures (Aiii and E). Numbers of cells attaching in cell culture plates was also significantly reduced (B). All experiments were repeated three times. (TIF) [file pone.0075538.s004.tif]

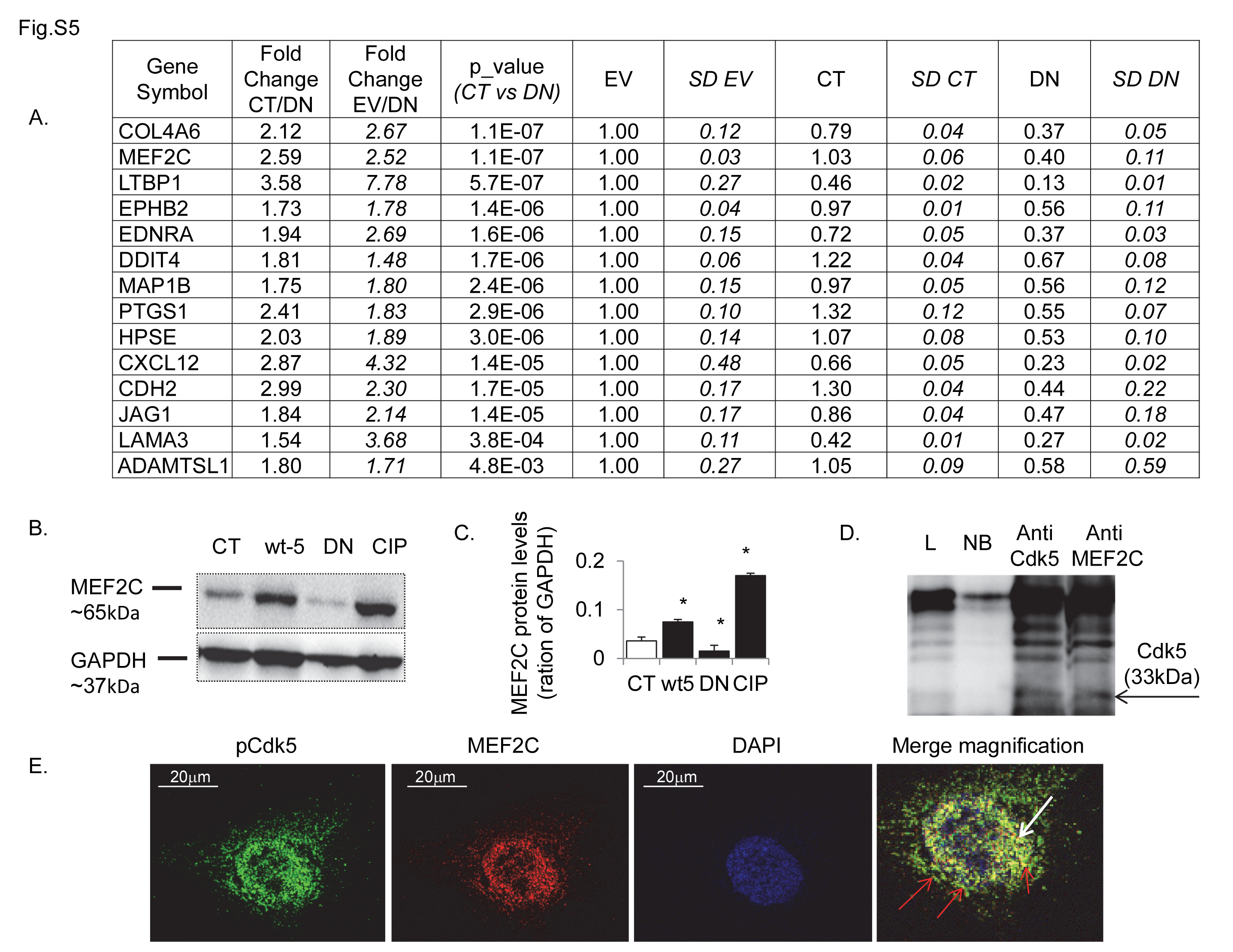

Supplement: Figure S5 — Gene microarray studies identified MEF2C down-regulation in Cdk5-DN mutants: (A) gene array results with Western blot confirmation of MEF2C protein down-regulation in DN mutants (B-C); (D) immunoprecipitation showing direct intracellular binding of Cdk5 with MEF2C protein and (E), double immunoflourescent labelling demonstrating MEF2C co-localization with phospho-Cdk5. All experiments were repeated three times. (TIF) [file pone.0075538.s005.tif]

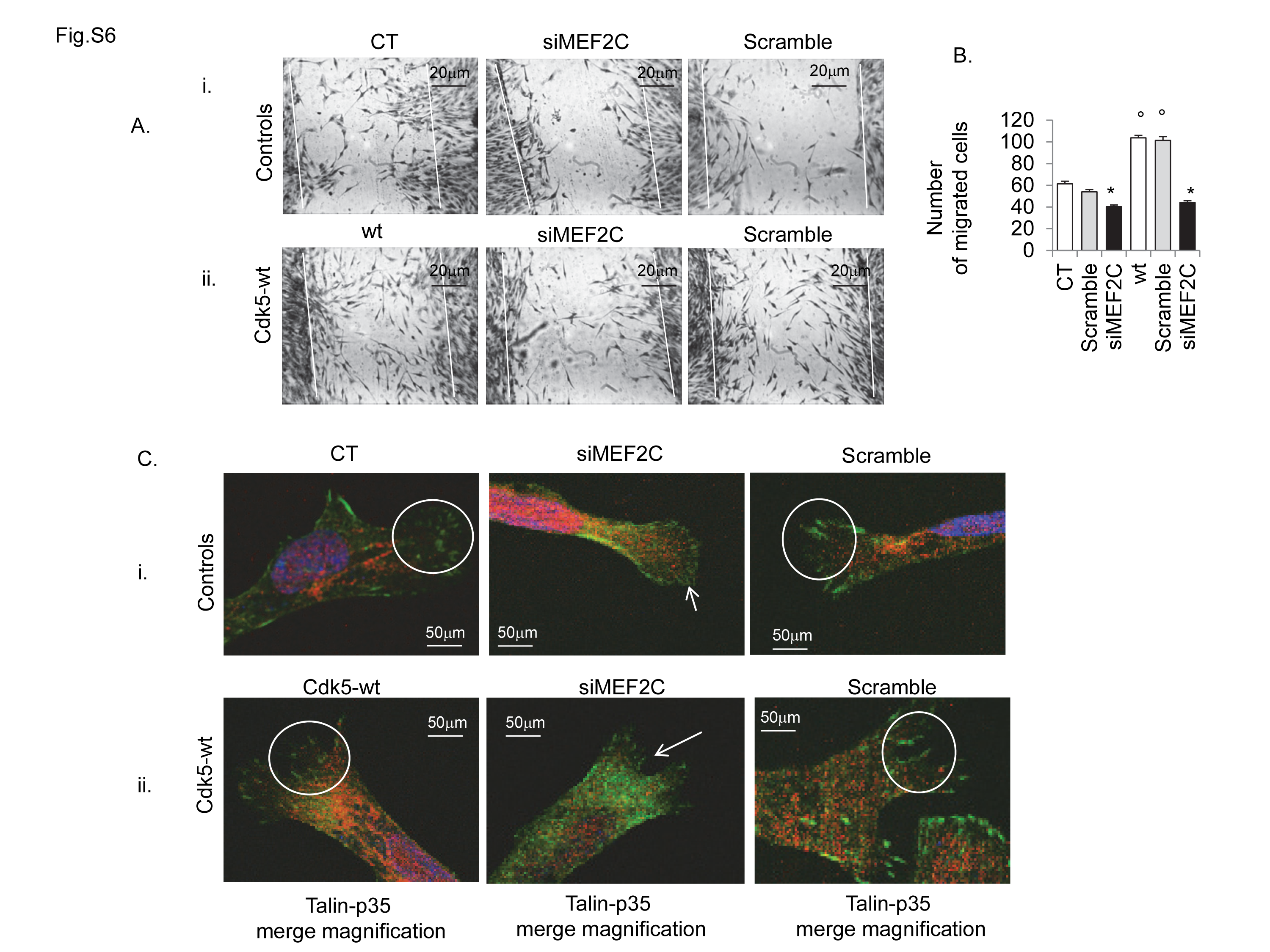

Supplement: Figure S6 — siRNA down-regulation of MEF2C inhibited hBMEC angiogenesis and talin-p35 co-localization in spreading cells: (A-B) siRNA to MEF2C significantly inhibited hBMEC migration in the scratch wound assay; (C) double immunoflourescent labelling showed notably reduced talin-p35 protein interaction at the tips of spreading cells concomitant with reduced ability to polarise and spread. All experiments were performed three times. (TIF) [file pone.0075538.s006.tif]

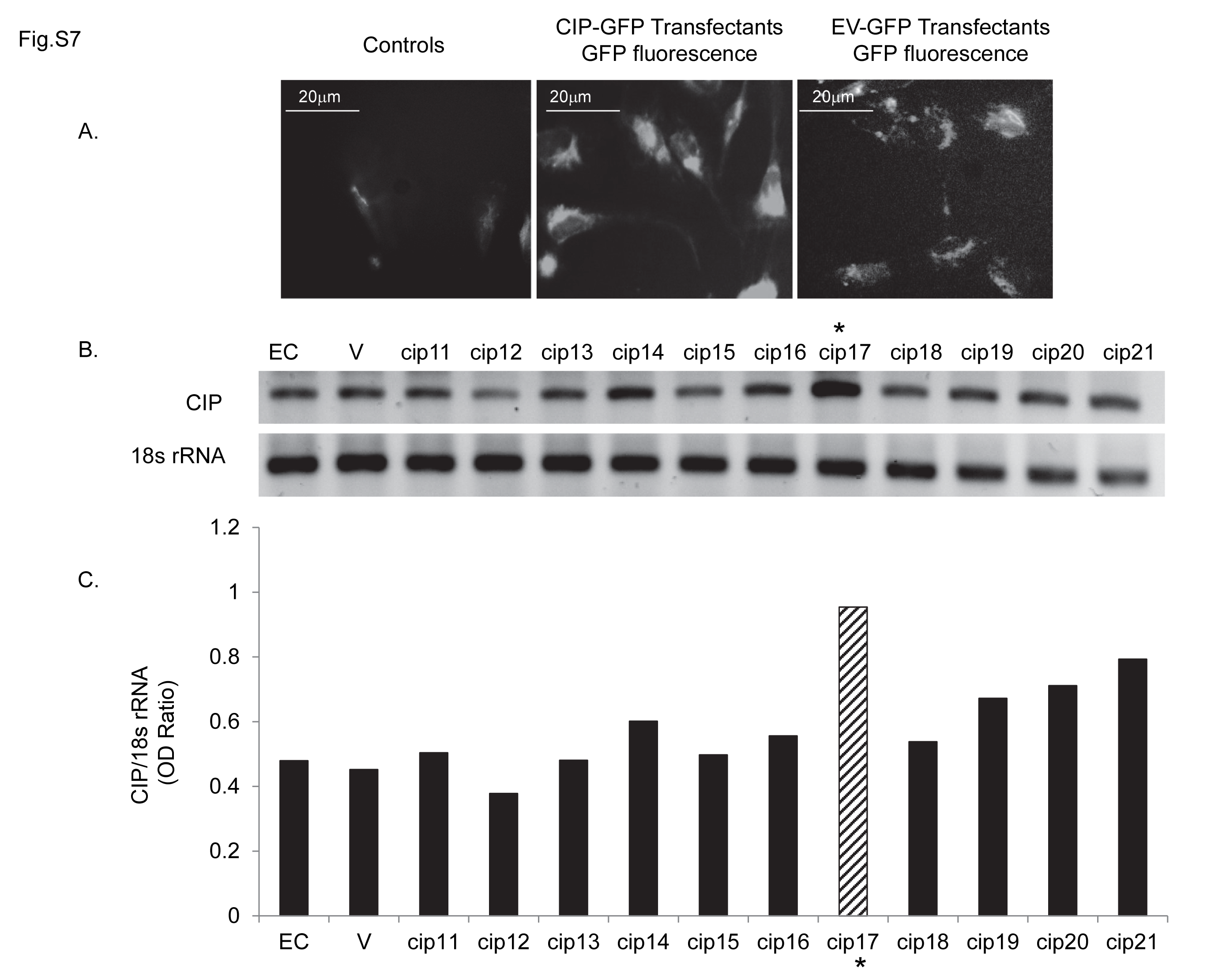

Supplement: Figure S7 — CIP transfectants expressed higher levels of CIP peptide: (A) immunofluorescent identification of CIP-GFP cellular uptake; (B-C) CIP transfectants e.g. CIP14 and CIP 17 expressed notably more peptide. (TIF) [file pone.0075538.s007.tif]

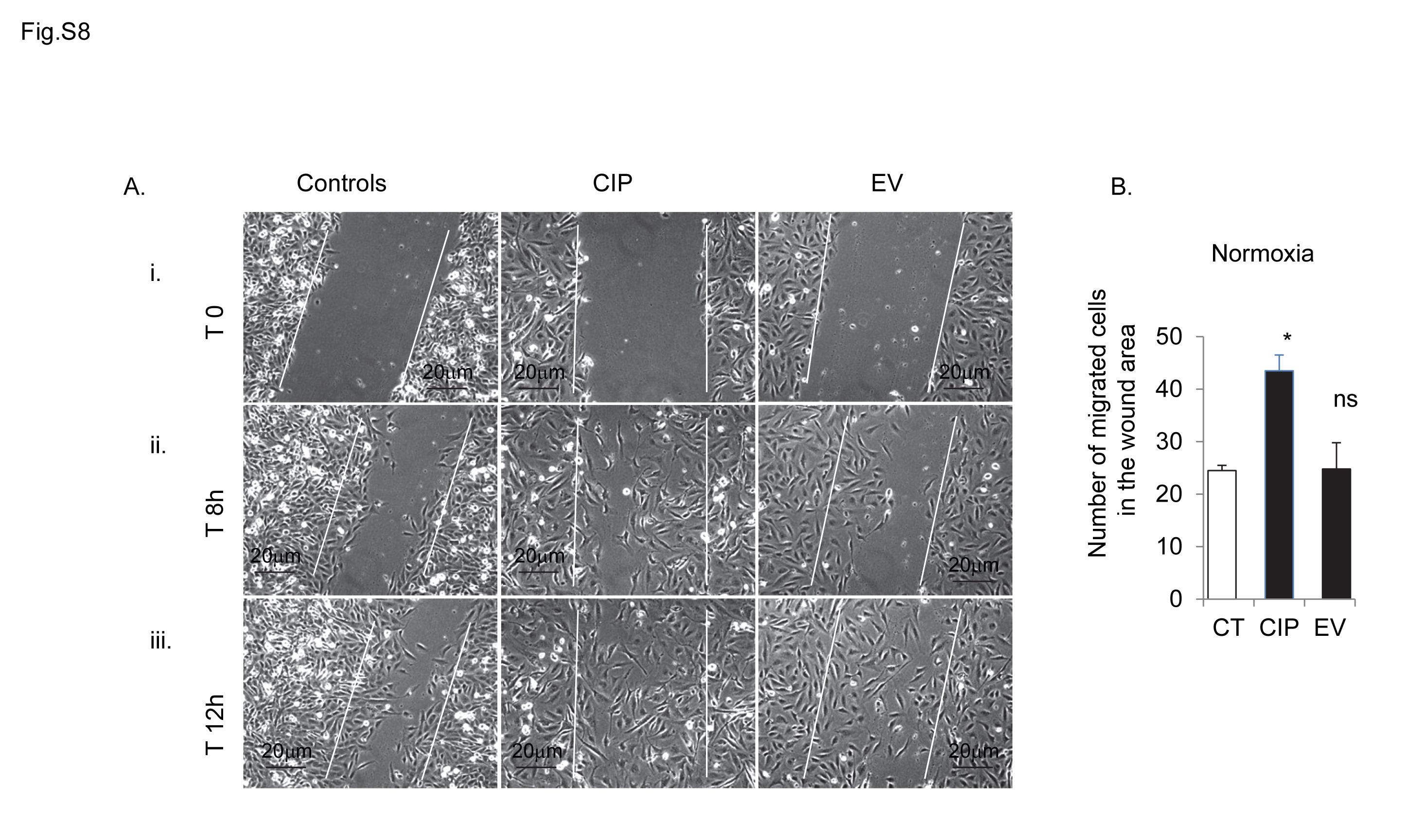

Supplement: Figure S8 — Effect of CIP transfection on hBMEC wound healing: (A-B) The presence of CIP-vector inside hBMEC significantly increased wound closure/migration compared with empty vector/control cells. Experiments were repeated three times. (TIF) [file pone.0075538.s008.tif]

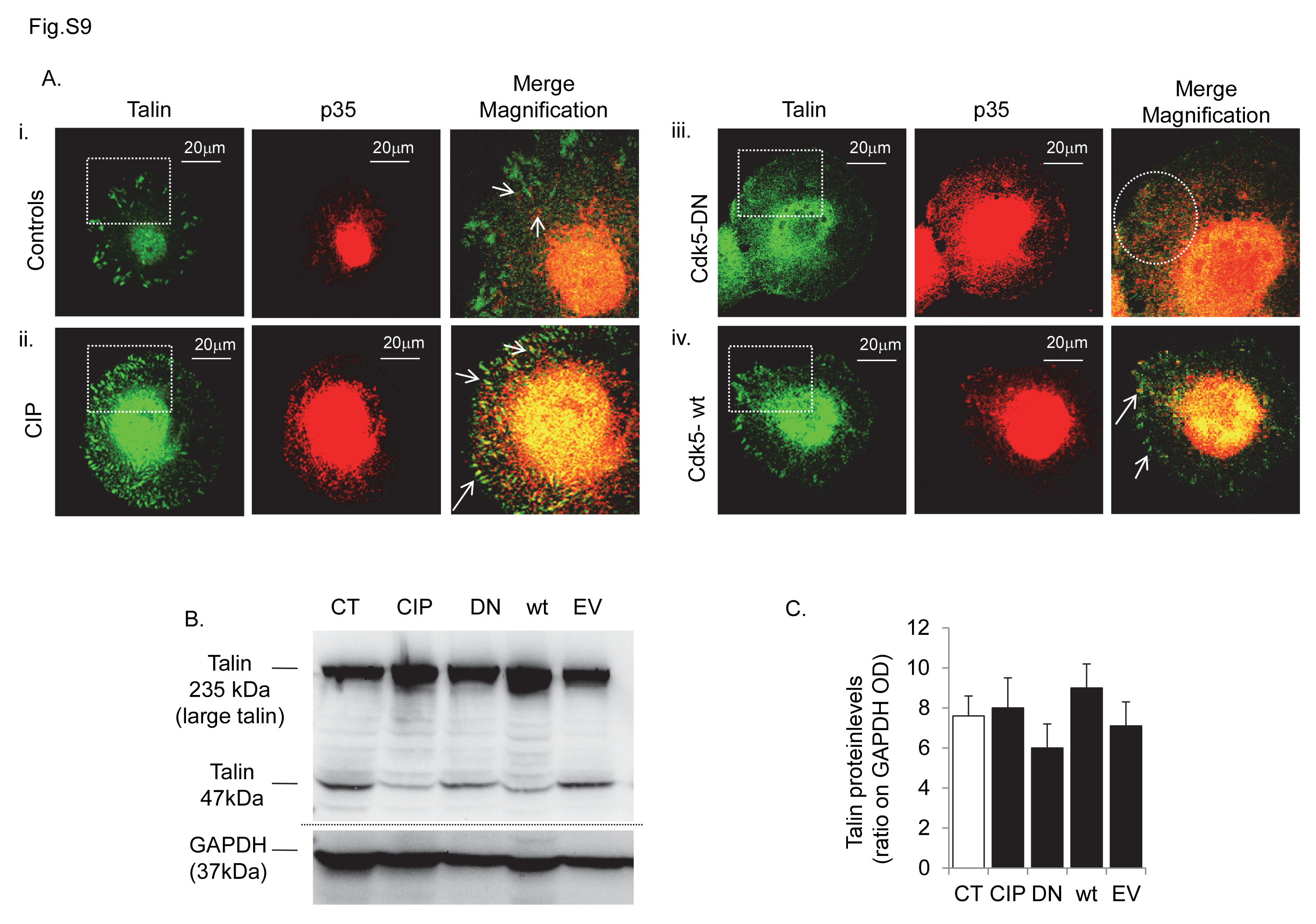

Supplement: Figure S9 — (TIF) [file pone.0075538.s009.tif]
